# Molecular mechanisms governing offspring metabolic programming in rodent models of in utero stress

**DOI:** 10.1007/s00018-020-03566-z

**Published:** 2020-06-03

**Authors:** Efthimia R. Christoforou, Amanda N. Sferruzzi-Perri

**Affiliations:** Department of Physiology, Development and Neuroscience, Centre for Trophoblast Research, University of Cambridge, Downing Site, Cambridge, UK

**Keywords:** Development, Programming, Metabolism, DOHAD, Fetal, Animal models

## Abstract

The results of different human epidemiological datasets provided the impetus to introduce the now commonly accepted theory coined as ‘developmental programming’, whereby the presence of a stressor during gestation predisposes the growing fetus to develop diseases, such as metabolic dysfunction in later postnatal life. However, in a clinical setting, human lifespan and inaccessibility to tissue for analysis are major limitations to study the molecular mechanisms governing developmental programming. Subsequently, studies using animal models have proved indispensable to the identification of key molecular pathways and epigenetic mechanisms that are dysregulated in metabolic organs of the fetus and adult programmed due to an adverse gestational environment. Rodents such as mice and rats are the most used experimental animals in the study of developmental programming. This review summarises the molecular pathways and epigenetic mechanisms influencing alterations in metabolic tissues of rodent offspring exposed to in utero stress and subsequently programmed for metabolic dysfunction. By comparing molecular mechanisms in a variety of rodent models of in utero stress, we hope to summarise common themes and pathways governing later metabolic dysfunction in the offspring whilst identifying reasons for incongruencies between models so to inform future work. With the continued use and refinement of such models of developmental programming, the scientific community may gain the knowledge required for the targeted treatment of metabolic diseases that have intrauterine origins.

## Developmental programming of metabolic disease

Metabolic syndrome was characterised by the World Health Organization (WHO) in 1998 as a disease diagnosed by the presence of insulin resistance in addition to two other criteria, namely obesity, hyperlipidemia, hypertension and microalbuminuria [1]. In modern societies, this non-communicable disease has risen in prevalence, with approximately one in four people being afflicted worldwide [2, 3]. Mutations in metabolic genes, as well as life-style factors such as calorie-dense diets and sedentary lifestyle are considered as main risk factors for developing the metabolic syndrome [4–6]. However, an often-overlooked risk factor for the development of metabolic syndrome, is the quality of one’s environment during gestation. Exposure to a suboptimal in utero environment due to a pregnancy complication or maternal nutrient deficit or surplus has been associated with the development of cardio-metabolic dysfunction in the offspring in later postnatal life [7–10]. This paradigm is known as the ‘Developmental origins of health and disease hypothesis’ and was first proposed by Barker and colleagues in 1986. Using birth and death records of men from Hertfordshire, England, Barker and others provided epidemiological evidence associating reduced birthweight, a surrogate for poor intrauterine nutrition and an increased rate of adult mortality from ischaemic heart disease [11]. These findings were also extended to demonstrate similar associations of poor intrauterine nutrition with adult rates of glucose intolerance and type 2 diabetes [12]. Studies of other human cohorts have since then also associated a variety of pregnancy complications such as maternal obesity, gestational diabetes and alcohol consumption to poor offspring metabolic health [13–15]. This association may be observed even when there is no birthweight change and may be exposed or exacerbated by unhealthy lifestyle choices, such as the consumption of a high calorie diet postnatally [16, 17].

The method by which early life exposures affect later metabolic health is thought to be mediated via epigenetic processes affecting the structure and function of offspring tissues. However, these processes are difficult to examine in a clinical setting and require the use of animal models. Animal models offer several advantages: gestational variables can be controlled or precisely altered, and the process of developmental programming studied in a comparatively shorter time than in humans. Indeed, numerous animal models of prenatal adversity have been developed in several species. Sheep, pigs, rabbits and guinea pigs are amendable to environmental manipulation and chronic instrumentation and previous reviews have highlighted their usefulness in studying developmental programming [18–21]. Mice and rats also offer advantages in the study of developmental programming due to the ease of maternal manipulation, availability of molecular tools (including gene manipulation) and short gestation and lifespan. However, rodents and humans vary in the temporal development of metabolic organs. For instance, white adipose tissue is present from birth in humans, but not rodents, even though brown adipose tissue is present in both. The development of the liver and the endocrine pancreas commences and completes earlier in gestation in humans than in mice [22–25]. Moreover, pancreatic beta cells are functional (i.e. exhibit glucose-stimulated insulin secretion) in humans from early gestation, whereas this only occurs in mice after birth [26–28]. However, many genes and signalling pathways governing the development of metabolic organs are shared between humans and mice. Moreover, several key maturational and metabolic events in fetal organs, such as a switch from a glycolytic to a gluconeogenic role in the liver occur around the time of birth in both species [24, 29]. In rodent metabolic organs that continue to develop and differentiate postnatally, environmental conditions during the lactational window can also contribute to the programming. Moreover, in the case of the white adipose tissue, which develops postnatally in rodents, alterations associated with gestational perturbations are likely secondary to programmed changes in the endocrine system occurring prenatally. The aim of this review is to summarise the findings from rodent models that show the involvement of changes in molecular pathways and the epigenetic mechanisms influencing these in offspring programmed for metabolic dysfunction as a result of an adverse in utero environment.

## Rodent models of developmental programming

Several models of developmental programming have been developed in mice and rats. The impact of maternal nutrient imbalance (total caloric restriction, high-fat diet and protein restricted diet), inhalation hypoxia, uterine ligation, stress (psychological), glucocorticoid exposure (dexamethasone administration), diabetes (streptozotocin treatment) and alcohol intake on offspring metabolic physiology are shown in Table 1. Of the reviewed studies, the majority were undertaken on male offspring. However, when offspring of both sexes were studied, sexual disparities in metabolism and molecular pathways with maternal manipulations have been identified (Table 1). These models have clinical relevance as undernutrition, specific nutrient deficiencies, obesity and diabetes are seen in women in both developed and developing countries and are associated with an increase in the propensity of their offspring to develop metabolic disease in later life [30–33]. In addition, hypoxia is a main cause of fetal growth retardation in women living at a high altitude, as well as those at sea level with sleep apnoea or exposed to environmental pollution or tobacco smoke [34–37]. Reductions in utero-placental blood flow can be seen in human pregnancy complications and despite the warnings of health impacts on the unborn child, alcohol may still be consumed by women during pregnancy [38, 39]. Finally, endogenous glucocorticoids are increased by maternal stress and synthetic glucocorticoids are administered to women with asthma or at risk of preterm birth [40–42]. However, regardless of the type of maternal insult during pregnancy, changes in offspring metabolism are linked to alterations in structure and molecular profile of key metabolic organs, namely the pancreas, liver, skeletal muscle and white adipose tissue (Table 1). Moreover, it is important to note that other maternal manipulations, including exposure to endocrine disruptors (bisphenol A), arsenite, nitric oxide synthase inhibitors (NG-nitro-L-arginine methyl ester), lipopolysaccharide, caffeine, vitamin D and iron deficiency, microbiota alterations, disruptions in the circadian rhythm, cigarette smoke and air pollutants (diesel exhaust particles) have also been linked to metabolic aberrations in rodent offspring, but are reviewed elsewhere [43–59].

**Table 1:** The effects of in utero stressors on molecular pathways in relation to offspring metabolic outcomes

Model (maternal manipulation)			Species		Sex studied	Treated from	Molecular alterations in metabolic tissues*unless otherwise stated, alterations occur in the sex studied	Metabolic outcomes	References
*Total calorie restriction*									
Undernutrition (10 g/day)			Rat		F	D1-23	2 months (muscle):↑ GLUT1, ↓ GLUT4	↓ plasma insulin, muscle glucose utilisation	[60]
Undernutrition (30%)			Rat		M&F	D1-23	8 months (liver): ↑ PKCζ	↑ plasma triglycerides	[61]
Undernutrition (30%)			Rat		M	D1-23	7 days (liver): ↑ *Pck1,* PEPCK, G6Pase, 7 days (pancreas): ↓ *Pdx-1, Nkx6-1, Pax6, Mafa, Ins1, Gcg* 21 days (adipose): ↑ *Dlk1, Cebpa, Pparγ, Srebf1, leptin, Adipoq, Ppargc1a, Adrb3* ↓ *Egr2, Klf5, Il1rn* 4 months (adipose): ↑ *Apelin* 4 months (liver): ↓ AKR1C21, ↑ CD36, G6PC 9 months (adipose): ↓ INSRβ, PI3K, PKCζ, mTOR	↑ adipocyte size, plasma apelin, ↓ glucose uptake in adipose, islet size, glucose tolerance	[62–65]
Undernutrition (30%) with postnatal HFD challenge			Rat		M		4 months (adipose): ↑↑ *Apelin,* ↑ *Apj*	↑ adiposity, plasma glucose, triglycerides, corticosterone, ↑ hepatic cholesterol, triglycerides	[64, 65]
Undernutrition (50%)			Rat		M&F	D1-23	0 days (pancreas): ↓ *Pdx-1, Insulin, Cacna1c, Cacna1d,* ↑ *Ghrelin* 3 weeks (pancreas): ↓ *Pdx-1, Insulin* 3 weeks (liver): ↑ *Lxr-α, Lpl* 3 months (pancreas): ↓ *Ucp2, Gck*, ↑ *Pparγ, Glut2, citrate synthase* (males) 3 months (liver): ↓ citrate synthase 5 months (liver): ↓ *Igfbp1* 5 months (adipose): ↓ pAKT, *Pgc1α, Pparγ, Pparα, Glut4, Irs1, InsR,* ↑ *Pten*	↓ ROS (females) and ATP content in islets, β cell mass and insulin secretion in response to glucose, plasma insulin, ↑ adiposity, adipocyte size and markers of inflammation in adipose, hepatic triglycerides, plasma ghrelin	[66–71]
Undernutrition (50%) with postnatal HFD challenge			Rat		M			↑↑ adiposity, markers of inflammation in adipose, ↑ plasma insulin and plasma leptin, insulin resistance	[71]
Undernutrition (50%)			Rat		F	D11-21	14 months (muscle): ↓ *Glut4*		[72]
Undernutrition (50%)			Rat		M	D10-21	1 day (liver): ↓ PPARα, PPARγ, ↑ Hepatic lipase, CRP 9 months (liver): ↓ PPARα, PPARγ, ↑ SREBP1, FAS	↑ hepatic triglycerides and plasma CRP	[73]
Undernutrition (50%)			Rat		M	D10-23	D20 (liver): ↑ SREBP1c, FAS 1 day (liver): ↑ SREBP1c, FAS, Hepatic lipase	↑ hepatic lipid content	[74]
Undernutrition (50%)			Rat		M	D14-PD21	3 weeks (adipose): ↑ *Npy, somatostatin, Pyy, Ucp1, Pgc1α, Pparα* ↓ *calcitonin-related peptide, galanin, Il6, leptin*	White adipose displays brown-like phenotype at weaning	[75]
*Low-protein diet*									
Protein restriction (8% vs 23%)			Rat		M	D1-PD21	2 months (liver): ↑ pAKT, GGT, GST-pi 1	↑ hepatic cholesterol, oxidative stress markers in liver ↓ hepatic glycogen	[76]
Protein restriction (8% vs 23%) with postnatal LPD challenge			Rat		M		2 months (liver): ↑ TGFβ ↓ IKβα ↑ GGT, GST-pi 1, pAKT, pERK	↓ liver weight, hepatic glycogen, ↑ hepatic cholesterol, triglycerides, serum pro-inflammatory cytokines, ↑↑ oxidative stress markers in liver	[76]
Protein restriction (8% vs 20%)			Rat		M&F	D1-PD21	3 weeks, 3 months, 11 months (liver): ↑ PEPCK, ↓ GCK	↑ plasma insulin (males)	[77, 78]
Protein restriction (8% vs 20%) with postnatal HFD challenge			Rat		M&F		3 months (liver): ↑ PEPCK, ↓ GCK	↑↑ plasma insulin (males)	[78]
Protein restriction (8% vs 20%)			Mouse		M&F	D1-PD21	3 weeks (liver): ↑ *Aqp7, Cpt1b, Lpl, Pparα, Pparγ,* ↓ *Cyp7a1*	↓ serum cholesterol, insulin sensitivity	[79]
Protein restriction (8% vs 20%)			Rat		F	D1-PD21	21 months (muscle): ↓ PKCζ 21 months (liver): ↑ INSRβ	↓ insulin sensitivity	[80]
Protein restriction (8% vs 20%)			Rat		M	D1-PD21	10 days (liver): ↑ *Igfbp1, Als* 3 weeks (liver): ↑ *Igf-1, Als* 3 months (adipose): ↑ INSR 3.5 months (muscle): ↓ *Sirt3* 4.5 months (adipose): ↑ *Fas, G3pd, Scd, Glut4, leptin,* ↓ *F-spondin, 12-Lpo, C/ebpα, Pparγ*	↑ adiposity, glucose tolerance, ↓ plasma insulin, bone mineral density, skeletal muscle mitochondrial oxidative respiration	[81–84]
Protein restriction (8% vs 20%) with postnatal LPD challenge			Rat		M			↓↓ bone mineral density, ↓ lean mass, ↑ serum IGFBP1	[82]
Protein restriction (8% vs 20%) with postnatal HFD challenge			Rat		M		3.5 months (muscle): ↑ *Ucp3, Sirt3*	↑ skeletal muscle mitochondrial oxidative respiration	[81]
Protein restriction (8% vs 20%)			Rat		M&F	D1-23	3 months (pancreas): ↑ *Ucp2* (males), *Glut2* (females)	↓ β cell mass, ↑ plasma triglycerides, islet ROS content (males for all parameters)	[85]
Protein restriction (8% vs 20%)			Rat		M&F	D1-23 or D1-PD21 or PD1-PD130	D19 (liver): ↓ CYP7A1 3 weeks (liver): ↓ CYP7A1 4 months (liver): ↓ CYP7A1	↑ plasma and hepatic cholesterol	[86]
Protein restriction (8% vs 20%)			Rat		M	D1-23	D20 (liver): ↑ TNFα, p-JNK, ↓ HNF4α, CYP7A1 Week 3 (liver): ↑ p-JNK, ↓ CYP7A1 3 months (pancreas): ↑ *p16, p21* 4 months (liver): ↓ *L*xr-α*,* ↑ G6Pase, 11β-HSD1 4.5 months (pancreas): ↓ PDX-1, Insulin, GLUT2 9 months (liver): ↑ TNFα, p-JNK, ↓ HNF4α, CYP7A1	↑ hepatic cholesterol, ↓ glucose tolerance	[87–90]
Protein restriction (9% vs 23%)			Mouse		M&F	D1-20	4 month (pancreas): ↓ *Pdx-1,* mTOR, pS6K	↑ plasma glucose ↓ β cell area, insulin secretion, glucose tolerance, insulin sensitivity	[91]
Protein restriction (9% vs 18%)			Rat		M&F	D1-23	1 month (liver): ↑ *Gr, Pepck, Pparα, Aox* 1 month (muscle): ↑ *Glut4* (females)	↓ muscle glycogen	[92–94]
Protein restriction (9% vs 18%)			Rat		M&F	D0-7, or D8-14 or D15-22	3 months (liver): ↓ SREBP-1c	↑ plasma insulin (males)	[95]
Protein restriction (9% vs 18%) with postnatal HFD challenge			Rat		M&F		3 months (liver): ↑ SREBP-1c	↓ liver size, (males & females) ↑ adiposity (males & females, but fat depot altered depends on offspring sex), plasma insulin (males)	[95]
Protein restriction (9% vs 17%)			Rat		M&F	D1-23	D22 (liver): ↑ *iNOS, eNOS*, ↓ *Insr, Igf1r, Igf2r*		[96]
Protein restriction (8% vs 17%)			Rat		M	D1-23	4 months (muscle): ↑ *Cpt1a, Pparα, Pgc1α, Ucp3*	↑ density of type II muscle fibres, ↓ rate of fatty acid oxidation, glycolysis in soleus	[97]
*High-calorie diet*									
High-fat diet (23%)			Rat		M&F	D1-23	3 months (pancreas): ↓ *Ucp2, Gck, Glut2* (females)	↑ β cell mass (females), ↓ ROS, ATP content in islets	[67]
High-fat diet (24%)			Rat		M	3 weeks prior, D1-PD21	12 months (muscle): ↓ p-AKT ↑ total AKT, ↓ mitochondrial complexes I, II, and V protein	↑ adiposity, plasma insulin	[98]
High-fat diet (24%)			Rat		M	D1-PD21	3 months (liver): ↓ *Gpx1, Sod1, Pon1, Pon2, Pon3, p16,* ↑ *Cox2* 5 months (liver): ↓ *Igfbp1*	↑ plasma insulin, leptin, IGF-1, IGFBP3, LDL, HDL, lipase, triglycerides, TBARS, hepatic lipid droplets	[68, 99]
High-fat diet (45%)			Rat		M&F	D1-PD21	2 days (liver): ↑ *Cdkn1a, Pmp22*, ↓ *Tbl1, Ccnf, Pcna, Ccna2, Mki67* 1 week (liver): ↓ *Wnt1*	↓ transition from G1 to S-phase in liver ↓ liver mass,	[100]
High-fat diet (45%)			Rat		M	D1-PD21	1 week (liver): ↓ *Wnt1*	↑ hepatic triglycerides	[101]
High-fat diet (45%)			Mouse		M&F	8 weeks prior to mating	D14.5 (adipose): ↑ *Zfp423*	↑ adipogenic differentiation	[102]
High-fat diet (49%)			Mouse		M	3 months prior, D1-PD21	6 months (pancreas): ↑ FOX01, Insulin ↓ IRS1, PI3K, pAKT, PDX-1, GLUT2	↑ plasma insulin, leptin, cholesterol, triacylglycerol, adiposity, mass of pancreatic β and α cells, ↓ glucose tolerance	[103]
High-fat diet (45%)			Rat		M	6 weeks prior, D1-PD21	4 months (pancreas): ↑ *Il1-1a, Csf3, Slfn4, Cxcl6, Gabrp, Cldn2, Mx2* 4 months (liver): ↓ INSRβ	↑ plasma glucose, ↓ insulin sensitivity	[104, 105]
High-fat diet (45%) with postnatal HFD challenge			Rat		M		4 months (pancreas): ↓ *Cd69, Csf1, Tnfaip2* 4 months (liver): ↓ p-PI2K, p-AKT	↑ plasma insulin, ↓ glucose tolerance, ↓↓ insulin sensitivity	[104, 105]
High-fat diet (45%)			Mouse		M	3 weeks prior, D1-PD21	1 month (muscle): ↑ p-JNK, p-IKK, PTP1B 1 month (adipose): ↑ p-JNK, p-IKK, PTP1B, ↓ a7nAChR, pJAK2, pSTAT3 1 month (liver): ↑ pJNK1, ↓ a7nAChR, pJAK2, pSTAT3, pCREB/β 2.5 months (liver): ↑ PEPCK 2.5 months (muscle): ↑ PEPCK, p-JNK, p-IKK 2.5 months (adipose): ↑ p-JNK, ↓ *Pparγ*	↑ adiposity, inflammatory markers ↓ hepatic glycogen, insulin sensitivity	[106, 107]
High-fat diet (45%) with postnatal HFD challenge			Mouse		M		2.5 months (liver): ← → PEPCK, ↓ p-JNK 2.5 months (muscle): ↑ PEPCK, p-IKK, PTP1B 2.5 months (adipose): ↑ PTP1B, ↓ *Cidec, Pparγ*	↑↑ adiposity ↓ hepatic glycogen, glucose tolerance, ↑ plasma leptin	[106, 107]
High-fat diet (58%)			Mouse		M&F	D1-PD21	3 weeks (liver): ↑ *Cd36, Aqp7, Cpt1b, Fabp2, Pparα, Pparγ* (males for all genes)	↓ glucose tolerance, insulin sensitivity ↑ serum cholesterol, hepatic steatosis (males for all parameters)	[108]
High-fat diet (59%)			Rat		M	4 weeks prior, D1-23	3 months (liver): ↓ INSRβ, IRS1, ↑ PKCζ 3 months (muscle): ↑ INSRβ, p85	↑ hepatic triglyceride content, adiposity ← → glucose tolerance, insulin resistance	[109]
High-fat diet (60%)			Mouse		M&F	D12-PD21	5 months (adipose): ↑ *leptin, Gcg* ↓ *Leptr, AdipoQ*	↑ plasma cholesterol, LDL cholesterol, triglyceride, FFA, leptin ↓ HDL cholesterol, adiponectin, insulin sensitivity	[110]
High-fat diet (60%)			Rat		M	8 wks prior, D1-PD21	6 months (liver): ↑ SCD-1	↑ adiposity, plasma TAG	[111]
High-fat diet (60%)			Rat		M or M&F	14 weeks prior, D1-PD21	3 weeks (adipose): ↓ *Pparγ, Srebp1,* ↑ *Lpl* 9 months (adipose): ↑ *leptin, Fas, Srebp1* and ↓ *Pparγ, 11βHsd1, Ob-Rb* (males), ↑ *C/Ebpα* and ↓ *Adipoq, Gr* (females)	↑ adiposity, plasma leptin, adipocyte hypertrophy and hyperplasia, plasma corticosterone and ↓ insulin sensitivity, glucose tolerance (males), ↓ brown adipose (females)	[112, 113]
High-fat diet (60%)			Rat		M	8 weeks prior, D1-PD21	1 day (adipose): ↑ C/EBPβ, PPARγ, SREBP1, FAS, HSL, ↓ C/EBPα, SIRT1, NCoR, SMRT, SRC1 9 months (adipose): ↑ C/EBPβ, PPARγ, SREBP1, FAS, SRC1, TIF2, ↓ C/EBPα, HSL, LPL, SIRT1, NCoR, SMRT	↑ plasma glucose, insulin, triglycerides, adiposity	[114]
High-fat diet (62%)			Mouse		M&F	D1-PD21	20 weeks (pancreas): ↓ *Pdx-1* (males) ↑ *Pdx-1* (females)	↓ insulin secretion in response to glucose, pancreatic insulin content, islet area, ↑ islet oxidative stress (males), ↑ insulin secretion in response to glucose, pancreatic insulin content, islet area (females)	[115]
High-fat diet (62%) with postnatal HFD challenge			Mouse		M&F			↑ hepatic triacylglycerol content, adipocyte area, markers of inflammation in adipose	[115]
*Uterine substrate supply*									
Uterine ligation			Rat		M	D18	D21, 1 week, 3.5 months (pancreas): ↑ MnSOD 2 weeks (pancreas): ↓ *Pdx-1*	↓ β cell mass, glucose stimulated insulin secretion, islet ATP production, ↑ islet ROS production	[116, 117]
Uterine ligation			Rat		M&F	D19	0 days (liver): ↓ *IGF1* 1 day (muscle): ↑ GLUT1, ↓ Ins-Rβ, *ΑCCα, ACS* 3 weeks (liver): ↓ *Igf1* 3 months (pancreas): ↓ *Pdx-1*	↓ liver weight, β cell mass, ↑ plasma glucose	[118–121]
*Hypoxia*									
Hypoxia (10%)			Rat		M	D15-20	3.5 months (Liver): ↓ AKT-1, AKT-2, PKCζ 3.5 months (Muscle): ↓ AKT-1, GLUT4	↑ plasma FFA	[122]
Hypoxia (12%)			Rat		M	D15-D21	12 months (Liver): ↓ G6Pase	↓ plasma glucose (males), insulin (females)	[123]
Hypoxia (12%)			Mouse		M	D15-20	2.5 months (Liver): ← → p-AKT, p-IRS, P-PKCθ 2.5 months (Muscle): ← → p-AKT, p-IRS, P-PKCθ	↑ adipocyte size, plasma insulin, leptin, triglycerides, FFA, ↓ insulin sensitivity	[124]
Hypoxia with postnatal HFD challenge		Mouse			M		2.5 months (Liver): ↓ p-AKT, ↑ p-IRS, P-PKCθ 2.5 months (Muscle): ↓ p-AKT, ↑ p-IRS, p-PKCθ	↑ intra-abdominal fat, adipocyte size, plasma insulin, FFA, ↓ insulin sensitivity	[124]
Hypoxia (intermittent, 21–12%)			Mouse		M	D1-18.5	3.5 months (Muscle): ↓ p-AKT	↑ adiposity, plasma triglycerides, cholesterol, FFA, ↓ insulin sensitivity	[125]
*Maternal excess glucocorticoids/psychological stress*									
Dexamethasone treatment			Rat		M	D14-20	5 days (liver): ↑ *Pepck, Gr* 1 week (pancreas): ↓ *Pdx-1, Maf-a, Neuro d1, Pax-6* 3 weeks (liver): ↑ *Pepck* 3 months (liver) ← → PEPCK 4 months (muscle): ↓ *Insr* 4 months (adipose): ↓ *Insr, Irs1* 4 months (liver): *Adipoq, Hk2* 4 months (pancreas): ← → *Pdx-1, Maf-a, Neuro d1, Pax-6* 6 months (adipose): ↑ *Gr,* ↓ *Lpl, Pparγ* 6 months (muscle): ↓ *Gr* 8 months (liver): ↑ *Pepck, Gr*	↓ β cell fraction, insulin secretion in response to glucose, glycogen storage in muscle	[126–130]
Dexamethasone treatment			Rat		M	D17-23	1 week (liver): ↑ *Pck1, G6pc*, G6Pase 3 weeks (adipose): ↑ *Dlk1, Cebpa, leptin, Adipoq*	↓ pancreatic islet size, insulin sensitivity, glucose tolerance	[62]
Dexamethasone treatment			Rat		M&F	D14-21	3 weeks (liver): ↑PEPCK (males)	↑ plasma adrenocorticotropic hormone, corticosterone, postprandial insulin-glucose ratio (males)	[131]
Dexamethasone treatment			Rat		M&F	D15-19	D20 (liver): ← → G6Pase, PEPCK 0 days (liver): ← → G6Pase, PEPCK 3 weeks (liver): ← → G6Pase, ↑ PEPCK 3.5 months (liver): ← → G6Pase, ↑ PEPCK	↓ glucose tolerance (females)	[132]
Dexamethasone treatment			Rat		M&F	D18-23	2 months (liver): ↓ *Pparγ, Pgc1a* (males), ↑ *Cd36* (females)		[133]
Dexamethasone treatment with postnatal HFD challenge			Rat		M&F		2 months (liver): ↓ *PPARγ* (males) ↑ *Cd36* (females)	↑ hepatic steatosis, ↓ plasma IGF1 (females)	[133]
Dexamethasone treatment			Rat		M&F	D13-23	6 months (adipose): ↑ *Gr,* ↑ *Pparα, 11βHsd1* (males)	↑ plasma fatty acid, markers of inflammation	[134]
Psychological stress by exposure to aggressive rat			Rat		M&F	D16-20	6 months (liver): ↓ *Pgc1α* (females) 6 months (adipose): ↓ *Atgl, Dgat2, 11βHsd1, leptin, Pepck* (females) 6 months (adipose): ↓ *Pepck* (males) 6 months (muscle): ↓ *Pparα* (females), ↓ *Gr* (males)	↑ plasma triglycerides (males) ↑ plasma insulin (females)	[135]
Psychological stress by exposure to variable stimuli (eg restraint, noise, swim, light)			Rat		F	D14-21	4 months (adipose): ↑ *Fas, Scd1* ↓ *Il6*	↓ plasma insulin ↑ insulin sensitivity	[136]
Psychological stress with postnatal HFD challenge			Rat		F		4 months (adipose): ↑ *Adpn*	↑ adiposity	[136]
Psychological stress by injection and single housing			Rat		M&F	D15-19	D20 (liver): ↑ G6Pase, Pepck	↑ hepatic glycogen	[137]
Psychological stress by restraint			Mouse		M&F	D8-20	1 month (liver): ↑ *Alt2, Dgat1, Pparγ,* 11βHsd1, Gr	↑ hepatic lipids	[138]
*Streptozotocin injection to induce maternal diabetes*									
Streptozotocin injection			Rat		M or M&F	Prior to mating	3 months (adipose):↑ GLUT4, INSRβ, ACC 5 months (liver): ↑ *G6pc & Pck1* (FOXO1 pathway) and *Pdk4, Cdkn1a, Gadd45a, Igfbp1, Hmox1,* ↓ *Srebf1* 2–7 months (muscle): ↓ INSR 2–7 months (adipose): ↓ INSR 4.5 months (muscle): ↓ GLUT4 (males) 4.5 months (adipose): ↓ GLUT4	↑ plasma glucose, insulin, adipocyte diameter, adipose glucose uptake ↓ glucose tolerance, insulin sensitivity	[139–142]
Streptozotocin injection with postnatal HFD challenge	Rat			M&F			4.5–7 months (adipose): ↓ GLUT4 7 months (adipose):↑ *mTFA* 7 months (muscle):↑ *mTFA*	↑↑ plasma glucose	[139]
Streptozotocin injection			Rat		NS	D0	3 months (pancreas): ↓ *Kir6.1, Cdk4, E2f1*	↑ plasma glucose	[143]
Streptozotocin injection			Rat		F	D0.5	7 months (adipose): ↑ *Tnfα*	↓ pancreatic islet area, glucose tolerance, ↑ plasma glucose	[144]
Streptozotocin injection with postnatal HFD challenge			Rat		F			↓↓ glucose tolerance	[144]
Streptozotocin injection			Rat		M	D6 and D12	4.5 months (pancreas): ↓ *Abcc8, Cav1.2, Cav2.3*	↓ insulin sensitivity, glucose tolerance, ↑ plasma insulin	[145]
Streptozotocin injection with postnatal HFD challenge			Rat		M		4.5 months (pancreas): ↓↓ *Cav1.2*	↓↓ insulin sensitivity, glucose tolerance	[145]
*Alcohol exposure*									
Alcohol exposure			Rat		M &/or F	D1-23	1 day (adipose): ↓ *leptin* 3 months (adipose): ← → leptin 3 months (muscle): ↓ GLUT4, ↓ insulin stimulated p-AKT, p-PDK1, p-PKCζ, ↑ PTEN, TRB3 3 months (liver): ↑ PTEN, TRB3 3.5 months (liver): ↑ PEPCK, PGC1	↑ pancreatic islet area ↓ food intake, pancreatic insulin content, insulin sensitivity, glucose tolerance	[146–150]
Alcohol exposure			Rat		M&F	4 days prior, D1-4	8 months (liver): ↑ *G6pc, Pck1, Ppargc1a,* ↓ *Gck* 8 months (adipose): ↑ AKT, p-AKT (males), ↓ AKT, p-AKT (females)	↑ fasting plasma glucose, insulin resistance, glucose intolerance, gluconeogenesis	[151]
Alcohol exposure with postnatal HFD challenge			Rat		M&F		8 months (liver): ↓ *Pck1* (females)	↓ glucose tolerance, insulin sensitivity, ↑ plasma insulin (males)	[151]
Alcohol exposure			Rat		M	D1-7 or D7-14 or D15-21	4 months (liver): ↑ PEPCK, G6Pase, SIRT2 ↓ Acetyl-FOXO1	↑ gluconeogenesis, ↓ glucose tolerance	[152]
Alcohol exposure			Rat		M	D13.5 and D14.5	7 months (adipose): ↑ pan-AKT	↑ plasma insulin, ↓ insulin sensitivity	[153]
Alcohol exposure			Rat		F	D11-20	D20 (liver): ↑ *G6pase, Acc, Fas, Srebp1c, Hnf4α, Foxo1,* ↓ *Igf1, Mttp*	↑ plasma IGF1, glucose	[154]
Alcohol exposure with postnatal HFD challenge			Rat		F		5.5 months (liver): ↑ *Igf1,* ↑ *Igf1r, Irs2, Glut2, G6Pase, Accα, Fas, Srebp1c, Pparα, Foxo1,* ↓ *Mttp*	↑ plasma IGF1, glucose, triglycerides, liver steatosis	[154]

Alterations in gene expression are indicated by italicisation, and alterations in protein abundance is not italicised. *Gestational age: mouse 20 days, rats 23 days*

11βHsd1, 11β-Hydroxysteroid dehydrogenase type 1; 12-Lpo, 12 lipoxygenase; a7nAChR, nicotinic acetylcholine receptor alpha7 subunit; Abcc8, ATP binding cassette subfamily C member 8; Accα, acetyl-coenzyme A carboxylase alpha; Acs, acetyl-coenzyme A synthetase; Adipoq, adiponectin; Adpn, adiponutrin; Adrb3, adrenoceptor beta 3; Akr1c21, aldo–keto reductase family 1, member C21; Αkt, protein kinase B; Als, acid labile subunit; Alt2, alanine aminotransferase 2; Aox, acyl-Coa oxidase; Apj, apelin receptor; Aqp7, aquaporin 7; Cacna1c, calcium voltage-gated channel subunit alpha1 C; Cacna1d, calcium voltage-gated channel subunit alpha1 D; Cav1.2, calcium voltage-gated channel subunit alpha1 C; Cav2.3, calcium voltage-gated channel subunit alpha1 E, Ccna2, cyclin A2; Ccnf, cyclin F; Cd36, Cd36 molecule; Cd69, Cd69 molecule; Cdk4, cyclin dependent kinase 4; Cdkn1a, cyclin dependent kinase inhibitor 1A; Cebpa, CCAT/enhancer binding protein alpha; C/ebpα, CCAAT-enhancer-binding protein alpha; C/ebpβ, CCAAT-enhancer-binding protein beta; Cidec, cell death inducing DFFA like effector C; Cldn2, Claudin 2; Cox2, cytochrome c oxidase subunit 2; Cpt1a, carnitine palmitoyltransferase 1A; Cpt1b, carnitine palmitoyltransferase 1B; Crp, C-reactive protein; Csf1, colony stimulating factor 1; Csf3, colony stimulating factor 3; Cxcl6, C-X-C motif chemokine ligand 6; Cyp7a1, cytochrome P450 family 7 subfamily A, polypeptide 1; D, embryonic day; Dgat1, diacylglycerol acyl transferase 1; Dlk1, delta like non-canonical Notch ligand 1; E2f1, E2F transcription factor 1; Egr2, early growth response 2; eNOS, endothelial nitric oxide synthase; Erk, extracellular receptor kinase; iNOS, inducible nitric oxide synthase; F, females; Fabp2, fatty acid binding protein 2; Fas, fatty acid synthase; Fox01, forkhead box protein O1; F-spondin, vascular smooth muscle cell growth-promoting factor; G3pd, glyceraldehyde-3-phosphate dehydrogenase; G6Pase, glucose-6-phosphatase; G6pc, glucose-6-phosphatase catalytic subunit; Gabrp, gamma-aminobutyric acid type A receptor subunit Pi; Gcg, glucagon; Gck, glucokinase; Ggt, Gamma-Glutamyltransferase 1; Glut, glucose transporter; Gpx1, glutathione peroxidase 1; Gr, glucocorticoid receptor; Gst-pi1, glutathione S transferase pi 1; HFD, high fat diet; Hk2, hexokinase 2; Hnf4α, hepatocyte nuclear factor 4 alpha; Hsl, hormone-sensitive lipase ortholog; Igf1, insulin like growth factor 1; Igf1r, insulin like growth factor 1 receptor; Igf2r, insulin like growth factor 2 receptor; Igfbp1, insulin like growth factor binding protein 1; Ikβα, NFKB inhibitor alpha; Ikk, I kappa B kinase; Il6, interleukin 6; Il1-1a, interleukin 1-1a; Il1rn, interleukin 1 receptor antagonist; Ins1, insulin 1; InsRβ, insulin receptor beta; Irs1, insulin receptor substrate 1; Irs2, insulin receptor substrate 2; Jak2, janus kinase 2; Jnk, c-jun N-terminal kinase; Kir6.1, potassium inwardly rectifying channel subfamily J member 8; Klf5, kruppel like factor 5; Leptr, leptin receptor; LPD, low protein diet; Lpl, lipoprotein lipase; Lxr-α, liver X-receptor alpha; Mafa, MAF bZIP transcription factor A; Mki67, marker of proliferation Ki-67; MnSOD, manganese-dependent superoxide dismutase; MtfA, mitochondrial transcription factor A; mTOR, mammalian target of rapamycin; Mttp, microsomal triglyceride transfer protein; Mx2, Mx dynamin like GTPase 2; NCoR, nuclear receptor corepressor; Neurod1, neuronal differentiation 1; Nkx6-1, NK6 homeobox 1; NPY, neuropeptide Y; NS, not stated; Ob-Rb, leptin receptor (long); p16, cyclin dependent kinase inhibitor 2A; p21, cyclin dependent kinase inhibitor; p85, phosphoinositide-3-kinase regulatory subunit; Pax6, paired box 6; Pck1, phosphoenolpyruvate carboxykinase; Pcna, proliferating cell nuclear antigen; PD, postnatal day; Pdk1, pyruvate dehydrogenase kinase 1; Pdk4, pyruvate dehydrogenase kinase 4; Pdx-1, pancreatic and duodenal homeobox 1; Pepck, phosphoenolpyruvate carboxykinase; Pgc1α, PPARG coactivator 1 alpha; PI3K, phosphatidylinositol 3-kinase; Pkcζ, protein kinase C zeta; Pkcθ, protein kinase C theta; Pmp22, peripheral myelin protein 22; Pon1, paraoxonase 1; Pon2, paraoxonase 2; Ppar, peroxisome proliferator-activated receptor; Ppargc1a, PPARG coactivator 1 alpha; Ptp1b, protein tyrosine phosphatase 1B; Pten, phosphatase and tensin homolog; Pyy, peptide YY; S6K, ribosomal protein S6 kinase; Scd-1, stearoyl-CoA desaturase-1; Sirt1, sirtuin 1; Sirt2, sirtuin 2; Sirt3, sirtuin 3; Slfn4, Schlafen 4; Smrt, silencing mediator for retinoid and thyroid hormone receptor; Sod1, superoxide dismutase 1; Src1, steroid receptor co-activator 1; Srebp1, sterol regulatory element-binding protein 1; Srebp1c, sterol regulatory element-binding protein 1c; Srebf1, sterol regulatory element binding transcription factor; Stat3, signal transducer and activator of transcription 3; Tbl1, transducing beta like 1 X-linked; Tgfβ, transforming growth factor beta; Tif2, transcriptional intermediary factor 1; Tnfα, tumour necrosis factor alpha; Tnfaip2, TNF alpha induced protein 2; Trb3, telomere repeat binding factor 3; Ucp, mitochondrial uncoupling protein; Wnt1, Wnt family member; Zfp423, zinc finger protein 423. M, males; M&F, males and females; ↑/↓ increase or decrease; ←  → unaltered; ↑↑/↓↓ effects exacerbated by a postnatal HFD/LPD

## Pancreatic insulin production

Offspring exposed to in utero stress frequently show an altered capacity to produce insulin. Hence, defects in the endocrine pancreas are thought to at least partially contribute to the programming of perturbed glucose handling in these offspring. For instance, rat offspring exposed to maternal undernutrition and protein restriction show decreased pancreatic insulin expression in line with their hypoinsulinaemic state [62, 66, 70, 90]. Moreover, offspring from maternal undernutrition and protein restriction show glucose intolerance at 3 weeks and 4 months, respectively [62, 90]. In pups from undernourished dams, the reduction in insulin expression can be seen as early as on day 0 (at birth) and may be due to enhanced pancreatic expression of *ghrelin* [66, 70], a known inhibitor insulin synthesis [155]. Conversely, adult offspring from mothers fed a high-fat diet have increased pancreatic insulin expression [103], in agreement with their hyperinsulinaemic profile [62, 156–163]. An upregulation of pancreatic insulin production may although be secondary to programmed changes in peripheral insulin sensitivity or hepatic insulin clearance in the offspring from high-fat diet-fed mothers ([164, 165] and described below).

In models of maternal nutrient perturbation, programmed changes in offspring insulin production may also be attributed to alterations in pancreatic islet mass. For instance, adult offspring exposed to a maternal high-fat diet in utero have an increase in pancreatic β cell mass in accordance with their elevated circulating insulin concentration [103]. Conversely, a reduction in β cell mass and proliferation has been observed in embryos and adult offspring exposed to protein restriction and undernutrition in utero [66, 91, 129, 166]. Decreased β cell mass is also observed in the offspring of mothers treated with dexamethasone or have diabetes (due to streptozotocin treatment) or uterine artery ligation during gestation [116, 129, 144]. In the case of maternal dexamethasone treatment, this is associated with diminished pancreatic insulin secretion [129]. Reductions in the expression of transcription factors important for β cell neogenesis, namely pancreatic and duodenal homeobox 1 (*Pdx-1*) and *Maf bZip transcription factor A* (*Maf-a*) have been observed in the fetus and adult offspring subjected to maternal protein restriction, undernutrition, uterine artery ligation and dexamethasone treatment [62, 66, 90, 91, 117, 119, 129, 166]. Similarly, other regulators of pancreatic islet cell development, namely *Nk6 homeobox 1* (*Nkx6-1*)*, paired box 6 *(*Pax6*) and *neuronal differentiation 1 *(*Neuro d1*) are downregulated in 7-day-old offspring exposed to maternal undernutrition or dexamethasone administration [62, 129]. Offspring of dams fed a high-fat diet also demonstrate changes in *Pdx-1* expression, although this is sex-dependent; females showed increased and males showed decreased pancreatic *Pdx-1,* with a concomitant increase or decrease in pancreatic islet area, respectively [115]. A similar sexual disparity is also observed in the offspring of maternal protein restriction, with only males displaying a decrease in pancreatic islet mass [85]. The expression of factors involved in protein synthesis, cell senescence and cell cycle control have also been implicated in the programming of the offspring pancreas. For instance, a reduction in the mechanistic target of rapamycin (mTOR) pathway, which regulates protein synthesis, as well as markers of cellular senescence, namely *cyclin dependent kinase inhibitor 1A* (*p21*) and *cyclin dependent kinase inhibitor 2A *(*p16*)*,* are upregulated in the pancreas of adult offspring exposed to maternal protein restriction during pregnancy [87, 91]. Moreover, in offspring from streptozotocin treated dams there is a reduction in the abundance of cell cycle regulators, namely *cyclin dependent kinase 4 *(*Cdk4*) and *E2f1* in the pancreas [143]. In these offspring from diabetic dams, a reduction in the major subunit of the ATP-sensitive potassium channel, *Kir6.2* is also observed alongside reductions in the expression of the ion channels *ATP binding cassette subfamily C member 8* (*Abcc8*)*, calcium voltage-gated channel subunit alpha1 C *(*Cav1.2*) and *calcium voltage-gated channel subunit alpha1 E *(*Cav2.3*) [143, 145].

Increased oxidative stress and reduced mitochondrial function are also implicated in the defective pancreatic formation and function in offspring programmed by an adverse gestational environment. For instance, the mitochondrial uncoupling protein, *Ucp2* and cellular energy (ATP) content of islets are decreased in offspring exposed to maternal undernutrition or a high-fat diet during gestation [67]. Interestingly, the reduction in *Ucp2* expression in response to maternal undernutrition is sex-dependent, with only males showing a downregulation in this gene. Despite this sexual-disparity in *Ucp2* expression, the islets of both male and female offspring failed to increase ATP content in response to glucose [67]. Islet ATP content is also reduced in the pancreas of offspring from rat mothers with uterine artery ligation; however, this is accompanied by increased abundance of the mitochondrial antioxidant enzyme, manganese superoxide dismutase (MnSOD) [116]. An increase in cytokines, such as *interleukin 1-1a *(*Il1-1a*) and *colony stimulating factor 3 *(*Csf3*) is also seen in the pancreas of offspring from a maternal high-fat diet [105]. There are also programmed changes in the ability of the pancreas to metabolise glucose as a result of a maternal dietary insult during pregnancy however the nature of this effect depends on the insult and offspring sex. For instance, the expression of the glucose transporter, *Glut2* is reduced in the islets of female offspring from mothers fed a high-fat diet, increased in female offspring from mothers fed a low-protein diet and elevated in male offspring from mothers undernourished during pregnancy [67, 85]. There are also alterations in the sensing and handling of glucose in the pancreas of offspring exposed to maternal undernutrition or alcohol intake in utero, as indicated by a reduction in *glucagon* and *glucokinase *(*Gck*) expression, respectively [62, 67]. Moreover, in islets of offspring exposed to a maternal high-fat diet, there are programmed changes in the abundance of insulin signalling pathway components, with reduced insulin receptor substrate 1(IRS1), phosphatidylinositol 3-kinase (PI3K) and phosphorylated protein kinase B (p-AKT) [103]. Thus, several molecular pathways involved in pancreatic development, insulin production and glucose handling are programmed by exposure to a suboptimal in utero environment, although the specific nature of effects can be partly dependent on offspring sex.

## Hepatic insulin sensitivity

As a site for glucose uptake, storage and production, the liver plays a vital role in maintaining whole body glucose homeostasis. As such, a reduction in hepatic insulin sensitivity is associated with the development of the metabolic syndrome. Hepatic insulin sensitivity can be indicated by the levels of the insulin receptor, its downstream signalling proteins and genes governing glucose and lipid handling. In rodents, maternal protein restriction or high-fat diet during pregnancy are associated with reduced liver expression of insulin receptor (INSR), IRS1 and insulin like growth factor 1 receptor (IGF1R) in the fetus and adult offspring [96, 104, 109]. There are also reductions in the abundance of AKT proteins (total and activated/phosphorylated) in the liver of offspring exposed to hypoxia during gestation that are exposed by feeding a high-fat diet postnatally [122, 124]. The levels of the INSR and phosphorylated AKT are instead elevated in the liver of offspring exposed to maternal protein restriction [76, 80], even though these offspring are insulin resistant [80]. Together, these findings illustrate that there can be a disconnect between hepatic and body insulin sensitivity. Moreover, changes in the insulin signalling pathway in the liver may serve to compensate for programmed defects in offspring metabolism as a result of an adverse gestational environment.

## Hepatic gluconeogenesis

Dysregulations in the pathways controlling hepatic gluconeogenesis are frequently implicated in the development of metabolic disease [167, 168]. For instance, there are alterations in the expression and activity of phosphoenolpyruvate carboxykinase (PEPCK) and glucose-6-phosphatase (G6Pase), which are two key enzymes involved in gluconeogenesis [169]. Increased expression of PEPCK and G6Pase by the liver is generally observed in rodent offspring of mothers exposed to undernutrition, protein restriction, alcohol, psychological stress, dexamethasone or streptozotocin treatment [62, 77, 78, 88, 93, 126, 127, 131, 132, 137, 140, 149, 151, 152, 154]. In some cases, the nature of the effect is determined by offspring sex (Table 1). For instance, PEPCK expression and concomitant hyperglycaemia are increased in male, but not female offspring exposed to dexamethasone in utero [131]. Furthermore, there are sex-dependent changes in the expression of hepatic gluconeogenic genes including the transcription factor *forkhead box o1* (*Foxo1*) in the offspring exposed to maternal high-fat diet, diabetes or alcohol [103, 140, 154]. In another study, female offspring exposed to alcohol prenatally showed increased hepatic gluconeogenesis in association with an elevated expression of genes including, *phosphatase and tensin homolog* (*Pten*) and *telomere repeat binding factor 3* (*Trb3*) [150]. Conversely, male offspring from mothers exposed to inhalation hypoxia have decreased hepatic gluconeogenic capacity (as indicated by G6Pase expression), in line with their hypoglycemic state [123]. Hepatic *glucokinase *(*Gck*) expression is, however, decreased in offspring of both sexes exposed to maternal alcohol intake or protein restriction during gestation [77, 78, 151]. Together, these data indicate that the programmed changes in offspring hepatic gluconeogenesis are dependent on the type of maternal manipulation, as well as offspring sex.

In addition to alterations in gluconeogenesis, proteins involved in glucose uptake/transport, namely protein kinase C (PKC) and glucose transporter (GLUT) [170, 171], are frequently altered in the liver of offspring subjected to a suboptimal intrauterine environment. For instance, PKCζ is increased in the liver of rodent offspring exposed to maternal undernutrition or high-fat diet, but is unchanged or decreased in those from hypoxic dams [61, 109, 122]. Moreover, offspring from hypoxic dams and that are fed a high-fat diet from weaning show elevated hepatic PKCθ abundance in accordance with their greater sensitivity to develop metabolic syndrome [124]. Similarly, offspring exposed to maternal alcohol intake during gestation and then fed a high-fat diet after weaning, show upregulated hepatic *Glut2* expression, with a corresponding increase in plasma glucose [154]. Overall, adverse in utero environments can program defects in liver glucose handling in the offspring with consequences for metabolic physiology.

## Hepatic lipid metabolism

In offspring exposed to suboptimal maternal environments including undernutrition, protein restriction, high-fat diet, hypoxia and dexamethasone treatment, hepatic and circulating lipids, such as cholesterol are increased [61, 68, 69, 73, 74, 76, 86, 89, 99, 108, 109, 111, 122, 124]. Dysregulated hepatic lipid metabolism and an increase in circulating lipids are associated in the development of metabolic disease. As such, key transcription factors involved in hepatic lipid metabolism, namely sterol regulatory element-binding protein 1 (SREBP1) and the peroxisome proliferator-activated receptor (PPAR) ligands (PPARα, PPARγ and PPARδ), are thought to play a critical role. Indeed, mice genetically deficient in PPARγ develop insulin resistance [172] and PPAR agonists such as rosiglitazone and pioglitazone are widely used in the treatment of type 2 diabetes [173]. Neonates and adults from undernourished or dexamethasone-exposed dams show reduced hepatic PPARγ expression [73, 133]. There is also a reduction in hepatic PPARα in offspring exposed to maternal undernutrition [73]. However, offspring exposed to maternal low-protein or high-fat diets or psychological stress display increased expression of both *Pparα* and *Pparγ* [79, 92, 108, 138]. The liver X receptor alpha (LXR-α) is a transcription factor that increases lipogenic enzyme expression and reduces gluconeogenesis [174, 175]. In the liver of offspring exposed to maternal protein restriction, *Lxr-α* is decreased in association with increased G6Pase expression [88]. However, in offspring exposed to maternal undernutrition, hepatic *Lxr-α* is instead increased and is associated with enhanced expression of the lipogenic gene, *Lpl* [69]. There is also an increase in the expression of lipolytic genes, namely *Srebp1c, fatty acid synthase *(*Fas*)*,* and *hepatic lipase,* in the liver of fetal and adult offspring exposed to maternal undernutrition or alcohol [73, 74, 154]. The increase in hepatic lipolysis is consistent with the elevated hepatic triglycerides and lipid content observed in these offspring [73, 74]. Conversely, a reduction in *Srebp1c* is observed in adult offspring exposed to a low-protein diet in utero regardless of sex, despite only male offspring displaying hyperinsulinemia [95]. In response to a maternal low-protein during gestation, there is a decrease in the expression of *cytochrome P450 family 7 subfamily A* (*Cyp7a1*), a gene involved in the metabolism of cholesterol, in the fetal liver [79, 86, 89]. Postnatally, hepatic *Cyp7a1* expression continued to be altered; however, the nature of the change was dependent on whether the maternal protein restriction was or was not extended into the lactational period; *Cyp7a1* expression was instead restored to normal or increased in the offspring of mothers fed a normal protein diet during lactation but remained decreased in offspring of mothers fed the low-protein diet in lactation [86]. Changes in hepatic cholesterol handling were likely responsible for the aberrant levels of cholesterol in the plasma and liver of these offspring, with most pronounced effects observed in those from mothers who were protein restricted both during gestation and lactation [86, 89]. Thus, a poor intrauterine environment can program defects in the lipid handling of the liver of offspring. Moreover, adverse environmental conditions that continue into the lactational period may propagate or amplify changes in offspring metabolic physiology that were programmed in utero.

## Hepatic inflammation

Impairments in lipid handling can be associated with the activation of inflammatory pathways in the liver that also contribute to the pathogenesis of metabolic disease. In offspring exposed to maternal protein restriction, activation of c-jun N-terminal kinase (JNK), a protein involved in inflammatory signalling is increased in the liver and linked to a decreased expression of *Cyp7a1* and hepatocyte nuclear factor 4α (HNF4α) [89]. An increase in JNK activation is also observed in the liver of offspring exposed to a maternal high-fat diet [106, 107]. Tumour necrosis factor alpha (TNFα) activates the phosphorylation of JNK [176, 177], and increased hepatic *Tnfα* is also seen in offspring exposed to maternal protein restriction [89]. This effect is observed as early as on embryonic day 20 in the fetus and maintained until adulthood [89]. Exposure to maternal dexamethasone treatment is also associated with activation of inflammatory pathways the offspring liver, with elevated expression of the inflammatory markers, *Cd36* and *Tnfα* observed postnatally [65, 133]. Activation of JNK phosphorylation also occurs through glucocorticoid signalling and as such, changes in glucocorticoid production and activity have been implicated in the development of insulin resistance [178]. Thus, its perhaps not surprising that in studies of maternal protein restriction, stress and dexamethasone treatment, the expression of the glucocorticoid receptor (*Gr*) and the enzyme involved in converting inactive cortisone to cortisol (*11βHsd1*), are increased in the liver (as well as adipose and skeletal muscle) of adult offspring in association with both enhanced inflammatory state and impaired glucose tolerance [88, 92, 93, 130, 135, 138]. Moreover, *11βHsd1* is up-regulated in the adipose of male offspring in response to maternal dexamethasone treatment [134] and intrauterine glucocorticoid over-exposure due to the administration of carbenoxolone, an inhibitor of placental 11βHDS2 causes hyperglycaemia in male offspring [179, 180]. Thus, adverse gestational environments can program changes in glucocorticoid signalling and inflammatory pathways in the liver of the offspring and contribute to impaired glucose homeostasis.

## Hepatic development

The upregulation of inflammatory markers in the offspring exposed to maternal high-fat diet or dexamethasone treatment is associated with the development of fatty liver (hepatic steatosis) in the offspring [99, 108, 133, 181]. The development of hepatic steatosis in offspring exposed to a maternal high-fat diet is also related to a reduction in the expression of antioxidant defence genes, such as *Cu/Zn superoxide dismutase *(*Sod1*) and *glutathione peroxidase-1 *(*Gpx1*) and an increase in genes involved in the cellular senescence pathway, such as *p16* [99]. Due to a poor environment during gestation, there may also be greater sensitivity of the offspring to develop diet-induced hepatic steatosis postnatally. Indeed, offspring exposed to maternal alcohol intake during pregnancy have a greater propensity to develop liver steatosis with a high-fat diet after weaning [154]. There are also alterations in liver morphology in response to maternal hypoxia, undernutrition and high-fat diet, with a reduction in offspring liver size being observed [182–184]. A maternal high-fat diet or diabetes (via streptozotocin treatment) is additionally associated with increased expression of the cell cycle inhibitor, *cyclin dependent kinase inhibitor 1A *(*Cdkn1*), and a reduction in the developmental gene, *Wnt1* in the liver of exposed offspring [100, 101, 140]*.*

Insulin-like growth factor 1 (IGF1) is produced by the liver and acts in an autocrine/paracrine manner to promote offspring growth [185, 186]. In offspring from mothers with uterine artery ligation, poor fetal and postnatal growth are associated with the decreased hepatic expression and circulating levels of IGF1 [121]. Conversely, in offspring exposed to a protein restriction, high-fat diet or alcohol prenatally, hepatic IGF1 and IGF acid-liable subunit (ALS), as well as circulating IGF1 are increased and likely drive the postnatal catch up growth seen [68, 82, 154]. Exposure to maternal protein restriction, undernutrition, a high-fat diet or diabetes in utero, is also associated with programmed changes in the expression of the IGF binding protein, *Igfbp1* in the offspring liver; however, the specific nature of the impact depends on the maternal insult and study age [68, 82, 140]*.* The IGF1 axis is also implicated in the regulation of insulin sensitivity and fat and carbohydrate metabolism [187, 188]. Thus, programmed changes in the IGF1 system as a result of poor intrauterine environment could contribute to growth and metabolic health outcomes in the offspring.

## Adipose lipid metabolism and morphology

Alterations in adipocyte differentiation and lipogenesis are associated with the development of metabolic disease and changes in PPAR expression are implicated in this pathogenic process [62, 189]. Reduced PPARγ expression is observed in the adipose tissue of rodent offspring as a result of maternal undernutrition and a high-fat diet during pregnancy [71, 112, 113]. The observed reduction in PPARγ expression is dependent on sex, with only male offspring from a maternal high-fat diet being affected [112]. However, PPARγ and PPARα expression are upregulated in the adipose tissue of offspring in other studies of maternal undernutrition and high-fat diet, as well as in response to protein restriction or dexamethasone treatment during gestation [62, 114, 134]. Moreover, the expression of PPAR target genes, namely *Srebp1, Fas, Lpl, hepatic lipase* and *stearoyl-CoA desaturase-1* (*Scd1*) are upregulated in the adipose of offspring exposed to maternal undernutrition, a high-fat diet or stress [84, 112–114, 136]. Variations in offspring adipose *Ppar* expression between studies may relate to the timing and specific nutritional manipulation utilised, the fat depot studied, the age and sex of the offspring, whether birth weight was reduced and/or followed by catch-up growth, as well as whether obesity and insulin resistance also ensued in the model. Nonetheless, changes in offspring adipose *Ppar* expression were associated with an increase adipocyte size [63, 71] and body adiposity in offspring exposed to maternal undernutrition, protein restriction or high-fat diet during gestation [71, 84, 106, 107, 112].

Through the interaction of PPARγ with CCAAT/enhancer binding proteins (C/EBPβ/ and C/EBPα), adipocyte differentiation occurs [190]. Consistent with an upregulation of PPAR expression and adiposity, *C/Ebpα* expression is increased in the adipose of offspring born from mothers fed high-fat diet during pregnancy [112, 114]. However, there is also an augmented expression of *zinc finger protein 423 *(*Zfp423*)*,* a transcription factor crucial in adipogenic lineage commitment in the adipose of fetuses derived from obese mothers fed a high-fat diet from prior to and during pregnancy [102]. Exposure to maternal undernutrition or dexamethasone treatment in utero is related to upregulated expression of the preadipocyte marker, *Dlk1,* in the white adipose tissue of offspring postnatally [62]. Collectively, these studies indicate that an insult during in utero development can advance white adipocyte differentiation in the offspring. Maternal undernutrition during pregnancy is also associated with the browning of white adipose tissue in offspring, as indicated by an increase in *mitochondrial uncoupling protein 1 *(*Ucp1*) and *neuropeptide Y *(*Npy*) expression [75]. Together, these data suggest that molecular pathways governing adipocyte differentiation and overall adipose morphology can be dysregulated in offspring exposed to in utero stressors.

Adipokines such as leptin and adiponectin are implicated in the control of lipid storage, as well as insulin sensitivity. Importantly, adipokines are shown to be altered in the adipose of offspring as result of an adverse environment in utero*.* For instance, offspring from mothers fed a high-fat diet show increased leptin and reduced adiponectin (*Adipoq*) expression in their white adipose tissue [110, 112]. Moreover, the expression of another adipokine, *apelin,* is increased in the white adipose of offspring exposed to undernutrition in utero and exacerbated by a postnatal high-fat diet [64]. Conversely, maternal undernutrition, alcohol exposure or dexamethasone treatment during gestation is associated with decreased *leptin* and increased *Adipoq* expression in the white adipose tissue of offspring [62, 75, 146]. Adipokines are also known to induce inflammation and contribute to the pathogenesis of metabolic disease [191]. Indeed, offspring from high-fat diet-fed or diabetic mothers show enhanced expression of inflammatory markers, like JNK and *Tnfα* in their white adipose tissue [106, 107, 144]. Thus, adipocyte development, adipokine production and inflammatory state are programmed by, and likely contribute to, the development of insulin resistance frequently observed in offspring subjected to a suboptimal intrauterine environment.

## Adipose and skeletal muscle insulin resistance

In addition to the liver, the adipose tissue and skeletal muscle respond to insulin and play a key role in maintaining glucose homeostasis in the body. Moreover, there are alterations in the insulin sensitivity of these tissues in the offspring as a result of maternal manipulation during gestation. For instance, maternal protein restriction during gestation is associated with increased InsRβ in the white adipose of adult offspring and increased glucose tolerance [83, 109]. Conversely, exposure to a maternal high-fat diet is associated with increased INSRβ and p85 in the skeletal muscle and unaltered insulin sensitivity in adult offspring [109]. Moreover, INSR levels are decreased in the muscle, liver and adipose tissue in offspring exposed to maternal dexamethasone, ethanol exposure, diabetes or a high-fat diet in utero*,* although glucose tolerance was not altered in some instances [63, 109, 120, 128, 139, 148]. Generally, a decrease in total and activated AKT are observed in the adipose and muscle of offspring exposed to maternal hypoxia, high-fat diet or undernutrition during pregnancy [71, 98, 122, 125]. Maternal alcohol intake during gestation is also linked to altered levels of both total and activated AKT in the adipose tissue of offspring; however, the specific effect is dependent on the offspring sex and resulting metabolic phenotype [151, 153]. As evident from the aforementioned studies, different imposed maternal manipulations can program alterations in skeletal muscle and white adipose tissue insulin signalling in both male and female offspring. However, how these molecular alterations relate to changes in glucose and insulin dynamics in the offspring in vivo*,* is less clear.

## Adipose and skeletal muscle glucose uptake

The skeletal muscle and adipose tissue are key metabolic organs involved in the uptake and storage of glucose through the action of insulin [171]. In offspring exposed to maternal undernutrition, protein restriction or alcohol during gestation, the abundance of PKCζ is decreased in the muscle and adipose [63, 80, 147]. Moreover, the mRNA expression, abundance and/or translocation of the insulin-sensitive glucose transporter, GLUT4, is reduced in the skeletal muscle and white adipose tissue of offspring exposed to undernutrition, hypoxia or alcohol *in* utero [60, 71, 122, 146, 147]. In the case of maternal undernutrition, this effect may be influenced by offspring sex [72]. Indeed, *Glut4* expression is specifically decreased in the muscle of female rats exposed to a maternal undernutrition or low-protein diet during gestation [60, 94]. However, GLUT4 abundance is decreased in the muscle of male rats from diabetic dams but increased in the adipose tissue [139, 141, 142]. The abundance of the insulin-independent glucose transporter, GLUT1 is increased in the skeletal muscle of offspring in response to maternal undernutrition or uterine ligation [60, 118]. Thus, it is evident that poor maternal environments during gestation program perturbations in the uptake of glucose by skeletal muscle and adipose tissue in the offspring; however, these effects may also be dependent on offspring sex.

In addition to glucose uptake, there are programmed changes in the mitochondrial capacity in the skeletal muscle and white adipose tissue of the offspring in response to a suboptimal intrauterine environment. For example, a maternal low-protein diet during pregnancy is associated with reduced *sirtuin protein 3* (*Sirt3*) expression and mitochondrial respiration in the skeletal muscle of offspring [81]. There is also an increase in *Ucp3* expression, a greater density of type II muscle fibres and a reduction in the rate of fatty acid oxidation in the soleus muscle of offspring exposed to a maternal low-protein diet [81, 97]. Expression of mitochondrial electron transfer complexes are reduced in the muscle of offspring from high-fat diet-fed dams [98]. Moreover, *Ucp1* is increased and mitochondrial biogenesis reduced (indicated by the lower expression of *PPARG coactivator 1 alpha, Pgc1a*) in the white adipose tissue of offspring from dams undernourished during gestation [71, 75]. A reduction in *Pgc1α* is also observed in the liver of female offspring from mothers exposed to stress [135]. Thus, poor maternal nutritional intake or stress during pregnancy can lead to programmed changes in the mitochondrial capacity of metabolic tissues in the offspring. Such changes in mitochondrial capacity would be expected to mediate alterations in metabolic fitness of the offspring.

## The role of epigenetic mechanisms in the programming of the fetus

The term epigenetics, meaning “above the gene”, is used to describe phenotypic modification as a result of changes in gene expression without an alteration to the DNA sequence [192]. In the context of the developmental programming field, epigenetic alterations serve as a useful mechanism for the fetus to adapt to a changing environment in utero. However, epigenetic alterations occurring during gestation may be detrimental after birth where the postnatal environment, including nutrient availability, may be different to that prenatally. The most described epigenetic alterations to gene expression include DNA methylation, posttranslational histone modification and small noncoding RNA. The involvement of changes in DNA methylation, histone modifications and small noncoding RNAs in mediating changes in the function of metabolic tissues in offspring programmed as a result of environmental manipulation during gestation are summarised in Tables 2, 3 and 4, respectively. However, it is important to note that the three types of epigenetic alterations are not distinct from one another. Instead, they often occur simultaneously and influence one another in the developmental programming of offspring metabolic phenotype.

**Table 2:** The effect of in utero stressors on DNA methylation in offspring tissues

Model (maternal manipulation)	Species	Sex	Treated from	Epigenetic alterations	Alterations in gene expression	Metabolic outcome	References
*Low-protein diet*							
Protein restriction (8% vs 20%)	Rat	M	3 weeks prior, D1-PD21	3.5 months (adipose): ↑ methylation at ICR of *Igf2/H19*	↑ *Igf2*	↑ adipose expansion, ↓ insulin sensitivity	[193]
Protein restriction (8% vs 20%)	Rat	F	D1-23	4.5 months (skeletal muscle): ↑ methylation at *Pgc1α* promoter	↓ *Pgc1α*	↓ glucose tolerance	[194]
Protein restriction (9% vs 18%)	Rat	M or M&F	D1-23	Day 0 (liver): ↑ methylation at the *H19/Igf2* ICR + 1 month (liver): ↓ GR1_10_ promoter DNA methylation, ↓ *Dnmt1*, ↓ methylation at *Pparα* promoter	↑ *Gr, Pepck, Pparα, H19, Igf2*	↑ gluconeogenesis	[93, 195, 196]
Protein restriction (9% vs 19%)	Rat	M	D1-19.5	D19.5 (liver): ↑ methylation at the *Lxr-α* promoter	↓ *Lxr-α*		[197]
Protein restriction (10% vs 22%)	Mouse	M	D1-PD21	6 months (adipose): ↓ methylation at *leptin* promoter	↓ *leptin*	↓ adiposity	[198]
*High-calorie diet*							
High-fat diet (16%)	Mouse	M	D1-PD21	↑ methylation of *Pparγ, Lxr-α*	↓ *Pparγ, Lxr-α*		[199]
High-fat diet (45%)	Mouse	M or M&F	8 weeks prior to mating	D14.5 (adipose): ↓ methylation of *Zfp423* promoter 3 weeks (adipose): ↓ methylation of *Zfp423* promoter	↑ *Zfp423*	↑ adipocyte differentiation, ↓ adipose expansion	[102, 200]
High-fat diet (45%)	Mouse	M&F	1.5 weeks prior, D1-PD21	2, 3, 4 months (adipose): ↓ methylation of *Pparγ, Fas, adiponectin* promoters	↑ *Pparγ, Fas, adiponectin*	↑ adipose expansion, ↓ insulin sensitivity	[201]
High-fat diet (45%)	Rat	M	D1-PD21	2 days (liver): ↓ methylation at specific CpG dinucleotides of *Cdkn1a* 3 months (liver): ↓ methylation of *Inpp5, Pik3c2b, Cbl, Lar, Pklr*	↑ *Cdkn1a, Pklr*	↓ liver size	[100, 202]
High-fat diet (60%)	Mouse	M&F	D12-PD21	5 months (adipose): ↓ methylation of *leptin*, ↑ methylation of *adiponectin*, *leptin receptor*	↑ *leptin,* ↓ *Leptr, AdipoQ*	↑ cholesterol, triglycerides, ↓ insulin sensitivity	[110]
High-fat diet (60%)	Rat	M	2 weeks prior, D1-PD21	3 weeks, 9 months (adipose): ↑ methylation of *Pparγ2* promoter	↓ *Pparγ2*	↑ plasma triglycerides, insulin	[113]
*Hypoxia*							
Hypoxia (12%)	Rat	M	D15-21	12 months (liver): ↑ methylation of histone H3 [K9] surrounding the *G6Pase* promoter	↓ *G6Pase*	↓ circulating glucose	[123]
Hypoxia (12% intermittent)	Mouse	M&F	D0.5–18.5	4 months (adipose): ↑ methylation of adiponectin gene promoter	↓ *adiponectin*	↑ leptin, ↓ insulin sensitivity (males)	[125]
*Streptozotocin injection*							
Streptozotocin injection	Rat	F	D0.5	7 months (adipose): ↓ methylation of *Tnfα* gene promoter	↑ *Tnfα*	↓ pancreatic islet area, glucose tolerance, ↑ plasma glucose	[144]
Streptozotocin injection	Rat	M	D6 and D12	4.5 months (pancreas): ↑ methylation of *Abcc8, Cav1.2, Cav2.3*	↓ *Abcc8, Cav1.2, Cav2.3*	↓ insulin sensitivity, glucose tolerance, ↑ plasma insulin	[145]
*Uterine substrate supply*							
Uterine artery ligation	Rat	M	D18	7 weeks (pancreas): ↓ methylation of *Fgfr1,* ↑ methylation of *Vgf, Gch1, Pcsk5*	↑ *Fgfr1* ↓ *Vgf, Gch1, Pcsk5*	↓ β cell function, insulin sensitivity	[203, 204]

Abcc8, ATP binding cassette subfamily C member 8; AdipoQ, adiponectin; Cav1.2, calcium voltage-gated channel subunit alpha1 C; Cav2.3, calcium voltage-gated channel subunit alpha1 E; Cbl, Cbl proto-oncogene; Cdkn1a, cyclin dependent kinase inhibitor 1A; D, embryonic day; Dnmt1, DNA methyltransferase; F, females; Fas, fatty acid synthase; Fgfr1, fibroblast growth factor receptor 1; G6Pase, glucose-6-phosphatase; Gch1, GTP cyclohydrolase 1; GR, glucocorticoid receptor; ICR, imprinting control region; Igf2, insulin like growth factor 2; Inpp5, inositol polyphosphate-5-phosphatase E; Lar, tyrosine-protein phosphatase Lar, Leptr, leptin receptor; Lxr-α, liver X-receptor alpha; M, males; M&F, males and females; Pcsk5, proprotein convertase subtilisin/kexin type 5; PD, postnatal day; PEPCK, phosphoenolpyruvate carboxykinase; PGC1α, PPARG coactivator 1 alpha; Pik3c2b, phosphatidylinositol-4-phosphate 3-kinase catalytic subunit type 2 beta; Pklr, pyruvate kinase L/R; PPARα, peroxisome proliferator-activated receptor alpha; PPARγ, peroxisome proliferator-activated receptor gamma; TNFα, tumour necrosis factor alpha; Vgf, VGF Nerve Growth Factor Inducible; Zfp423, zinc finger protein 423; ↑/↓ increase or decrease; Gestational age: mouse 20 days, rats 23 days

**Table 3:** The effect of in utero stressors on histone modifications in offspring tissues

Model (maternal manipulation)	Species	Sex	Treated from	Epigenetic alterations	Alterations in gene expression	Metabolic outcome	References
*Total calorie restriction*							
Undernutrition (50%)	Rat	F	D11-21	15 months (muscle): ↓ acetylation of H3K14	↓ *Glut4*	↓ insulin sensitivity	[72]
*Low-protein diet*							
Protein restriction (8% vs 20%)	Rat	M	D1-PD21	3 months, 15 months (pancreas): ↓ H3K4me1, ↑ H3K9me2 at *Hnf4α* enhancer	↓ *Hnf4α*	↓ glucose tolerance	[213]
Protein restriction (8% vs 20%)	Rat	M&F	D1-23 or D1-PD21	3 weeks (liver): ↑ methylation of histone H3 lysine 9 [K9, 14] at *Cyp7a1* promoter	↓ *Cyp7a1*	↑ circulating cholesterol	[86]
Protein restriction (8% vs 20%)	Rat	M	3 weeks prior, D1-PD21			↑ adiposity	[214]
Protein restriction (8% vs 20%) with postnatal HFD challenge	Rat	M		5 months (adipose): ↑ histone methyl transferase (G9a)	↓ *Fgf21*	↑↑ adiposity, ↓ insulin sensitivity	[214]
Protein restriction (9% vs 18%)	Rat	M&F	D2-23	1 month (muscle): ↑ acetylation of histone H3 histone H4 at *C/Ebpβ* promoter (females), ↑ acetylation of histone H3 histone H4 at *Glut4* promoter (females)	↑ *C/Ebpβ* (females), ↑ *Glut4* (females)	↑ glycogen content (females)	[94, 215]
*High-calorie diet*							
High-fat diet (45%)	Rat	M&F	D1-20	D21 (liver): ↓ association of H3Ac H3K4Me2, H3K9Me3, H3K27Me3 at *Pck1* promoter	↑ *Pck1*	↑ plasma glucose	[216]
High-fat diet (45%)	Rat	M&F	D1-PD21	1 week (liver): ↓ acetylation of H4 histone at the *Wnt1* promoter 3 months (liver): ↓ association of H3K9Me3 and ↑ association of H3K27Me3 (females) at *Pepck* promoter	↓ *Wnt1,* ↑ *Pepck* (females) ↓ *Pepck* (males)	↑ liver triglycerides	[101, 217]
High-fat diet (60%)	Mouse	M&F	4 months prior, D1-PD21	D18.5 (liver): ↑ lysine aceltransferase, ↓ histone deacetylase	↓ *Gck*		[218]
High-fat diet (60%)	Rat	M	16 weeks prior, D1-PD21	9 months (adipose): ↑ methylation, histone modification H3ac at *Pparγ2* promoter	↓ *Pparγ2*	↑ serum triacylglycerol, insulin, corticosterone	[113]
High-fat diet (62%)	Mouse	F or M&F	4 weeks prior, D1-20	2 weeks, 5.5 months (adipose): ↓ acetyl H3-K9 ↑ dimethyl H3-K9 at the *adiponectin* promoter, ↑ monometyl H4-K20 at the *leptin* promoter 2 weeks, 5.5 months (adipose): ↓ acetyl H3K9 levels ↑ dimethyl H3K9 in *adiponectin* promoter, ↑ monomethyl H4K20 in *leptin* promoter	↓ *adiponectin,* ↑ *leptin*	↓ insulin sensitivity, ↑ plasma triglycerides	[219, 220]
*Uterine substrate supply*							
Uterine artery ligation	Rat	M	D18	D21, 2 weeks, 6 months (pancreas): ↓ acetylation of H3 and H4 histones at the *Pdx-1* promoter	↓ *Pdx-1*	↓ insulin secretion, insulin sensitivity	[116, 117, 203, 221]
Uterine artery ligation	Rat	M&F	D19	0 days, week 3 (liver): ↓ me3K36 at H3 of *Igf1*	↓ *Igf1*	↓ insulin sensitivity	[121]
*Ethanol exposure*							
Ethanol exposure	Rat	F	D1-23	3 months (muscle): ↓ acetylation of *Pten, Trb3* 3 months (liver): ↓ acetylation of *Pten, Trb3*	↑ *Pten, Trb3*	↑ gluconeogenesis, ↓ insulin sensitivity	[147, 150]

Gestational age: mouse 20 days, rats 23 days

C/Ebpβ, CCAAT-enhancer-binding protein beta; Cyp7a1, cytochrome P450 family 7 subfamily A; D, embryonic day F, females; Fgf1, fibroblast growth factor 1; Gck, glucokinase; GLUT4, glucose transporter 4; HFD, high fat diet; Hnf4α, hepatocyte nuclear factor 4 alpha; Igf1, insulin like growth factor 1; M, males; M&F, males and females; Pck1, Phosphoenolpyruvate Carboxykinase 1; PD, postnatal day; Pdx-1, pancreatic and duodenal homeobox 1; Pepck, phosphoenolpyruvate carboxykinase; Pparγ2, peroxisome proliferator-activated receptor gamma 2; Pten, phosphatase and tensin homolog; Trb3, telomere repeat binding factor 3; Wnt1, Wnt family member 1*;* ↑/↓ increase or decrease

**Table 4:** The effect of in utero stressors on non-coding RNA expression in offspring tissues

Model (maternal manipulation)	Species	Sex	Treated from	Epigenetic alterations	Non-coding RNA targets	Metabolic outcome	References
*Total calorie restriction*							
Undernutrition (50%)	Rat	F	D11-21	3 weeks (liver): ↓ miR-122	↑ *Aldo-a, Bckdk, Pparβ, Pgc1α, Cpt1α*, ↓ *Fas, Hmgcr*	↑ fatty acid oxidation, ↓ fatty acid synthesis	[228]
*Low-protein diet*							
Protein restriction (8% vs 20%)	Rat	M	D1-PD21	D21 (pancreas): ↑ miR-375 10 days (pancreas): ↓ *H19* 22 days (adipose): ↑ miR-483-3p 3 months (pancreas): ↑ miR-375 3 months (adipose): ↑ miR-483-3p	↓ *Pdk1*, ↓ *Gdf3,* ↑ Let-7a	↓ insulin production, secretion ↓ glucose tolerance, ↓ adipocyte differentiation	[229–231]
Protein restriction (10% vs 23.5%)	Mice	M	D1-PD21	3 weeks (liver): ↓ mmu-miR-615, mmu-miR-124, mmu-miR-376b, mmu-let-7e, ↑ mmu-miR-708, mmu-miR-879	↑ *Il-6, Tnfα*	↑ inflammation	[232]
*High-calorie diet*							
High-fat diet (20%)	Mice	M	D1-PD21	2 months (adipose): ↑ miR-126	↓ *Irs1*	↑ serum insulin, ↓ insulin sensitivity	[233]
High-fat diet (23%)	Mice	F	4 weeks prior, D1-PD21	4 months (liver): ↓ miR-709, miR-122, miR-192, miR-194, miR- 26a, let-7a, let7b and let-7c, miR-494, miR-483	↑ *Pparα, Cpt1a*	↓ hepatic triglycerides, ↑ fatty acid oxidation	[234, 235]
High-fat diet (45%)	Mice	M	3 weeks prior, D1-20	0 days (liver): ↓ miR-122, ↑ miR-370 1 month (liver): ↓ miR-122, ↑ miR-370 2.5 months (liver): ↓ miR-122, ↑ miR-370	↓ *Acadvl, Cpt1,* ↑ *Agpat1, Gpam*	↑ hepatic triglyceride accumulation, ↓ fatty acid oxidation	[236, 237]
High-fat diet (58%)	Mice	M	D1-PD21	3 weeks (liver): ↓ miR-615-5p, miR-3079-5p, miR-124*, miR-101b*, ↑ miR-143*	↑ *Tnfα, Mapk1*	↑ inflammation, ↓ insulin sensitivity	[238]
High-fat diet (60%)	Mice	M&F	10 weeks prior, D1-20	D18.5 (adipose): ↑ miR-204-5p	↓ *Pgc1α, Sirt1*	↓ brown adipose tissue development, ↓ mitochondriogenesis	[239]

Gestational age: mouse 20 days, rats 23 days

Acadvl, Acyl-CoA Dehydrogenase Very Long Chain; Agpat1, 1-acylglycerol-3-phosphate O-acyltransferase 1; Aldo-a, Aldolase, F, females; Fructose-Bisphosphate A; Bckdk, Branched Chain Keto Acid Dehydrogenase Kinase; Cpt1a, Carnitine Palmitoyltransferase 1A; D, embryonic day; Fas, fatty acid synthase; Gdf3, growth differentiation factor 3; Gpam, Glycerol-3-Phosphate Acyltransferase, M, males; M&F, males and females; Mitochondrial; Hmgcr, 3-Hydroxy-3-Methylglutaryl-Coenzyme A Reductase; Il-6, interleukin 6; Irs1, insulin receptor substrate 1; Mapk1, mitogen-activated protein kinase 1; PD, postnatal day; Pdk1, pyruvate dehydrogenase kinase 1; Pgc1α, PPARG coactivator 1 alpha; Pparα, peroxisome proliferator activated receptor alpha; Pparβ, peroxisome proliferator activated receptor beta; Sirt1, sirtuin 1; Tnfα, tumour necrosis factor alpha; ↑/↓ increase or decrease

## Alterations in DNA methylation in response to in utero stressors

DNA methylation occurs when methyl groups are added to DNA, usually on the 5′-position of cytosine residues of a CpG island. The resulting heterochromatin condensation from DNA methylation is thought to be associated with a decrease in gene transcription [205]. However, DNA methylation is more complex than this, with increased methylation also associated with increased gene expression [206]. Alterations in DNA methylation are catalysed by the DNA methyltransferase enzymes (DNMTs), of which there are three types, DNMT1, DNMT3A and DNMT3B [207, 208]. The most studied epigenetic alteration in the developmental programming field is the methylation level of promoters of genes involved in lipid and glucose handling (Table 2).

Alterations in the methylation status of genes involved in hepatic development have often been reported in offspring exposed to nutrient perturbations during gestation. For instance, neonates from high-fat diet-fed mothers who subsequently showed reduced hepatic size and growth, displayed hypomethylation and increased expression of *Cdkn1a* in their liver [100]. Moreover, offspring from dams fed a low-protein diet during gestation showed increased methylation of the *Igf2/H19* imprinting control region (ICR) in line with enhanced *Igf2* expression in the liver. This was related to increased *H19* and *Igf2* expression in these day 0 male offspring [196]. Another study which showed a similar alteration in the *Igf2/H19* ICR upon maternal low-protein diet also illustrated insulin resistance and increased adipose expansion in offspring, implicating modifications in *Igf2/H19* methylation in adipose growth [193]. Moreover, alterations in the methylation of metabolic genes, including those for the insulin signalling pathway, such as PI3Ks, occur in the liver of offspring exposed to a maternal high-fat diet [202]. Offspring of mothers exposed to a low-protein diet during gestation show decreased methylation, associated with lower *Dnmt1* expression and increased hepatic *Gr* expression [93]. This reduction in *Gr* promoter methylation can also be observed in the grand-offspring (F2 generation) [209]. A maternal low-protein diet during pregnancy is also associated with the hypermethylation of the promoter for the *Lxr-α* gene, which encodes a nuclear receptor involved in fatty acid and cholesterol metabolism in the fetal liver [197]. Moreover, hypoxia during pregnancy causes modifications of histone H3, which surrounds the *G6Pase* gene promoter and is associated with a reduction of *G6Pase* levels in offspring with metabolic consequences, such as decreased circulating glucose [123]. In addition to alterations in glucose metabolism, fetal nutrient and oxygen deprivation achieved through maternal uterine artery ligation also has an impact on the pancreatic islets of offspring, with altered DNA methylation being linked to changes in the expression of genes involved in insulin secretion, islet proliferation and reduced islet mass [203, 204]. In the pancreas of offspring from diabetic mothers a reduction in the ion channels *Abcc8, Cav1.2* and *Cav1.3* is associated with the hypomethylation of these genes [145].

Alterations in DNA methylation also dictate the expression of genes involved in lipid metabolism in offspring born to mothers with suboptimal nutrition during pregnancy. A high-fat diet in utero causes epigenetic alterations in offspring adipocytokine genes in the visceral white adipose depot, with the hypermethylation of adiponectin and leptin receptor and hypomethylation of leptin observed. Such alterations in methylation of adiponectin and leptin receptors are associated with increased plasma triglycerides and cholesterol [110]. Conversely, exposure to a maternal low-protein diet in utero is associated with a decrease in methylation of the leptin gene promoter in adipose tissue [198]. Gestational hypoxia also causes hypermethylation of the adiponectin gene promoter with concomitant decrease in adiponectin levels in male offspring, who are more susceptible to metabolic syndrome in adulthood, as indicated by hyperleptinaemia and decreased insulin sensitivity. It additionally alters the methylation patterns of genes involved in metabolic regulation, including methionine degradation and cysteine biosynthesis in white adipose tissue of offspring exposed to maternal hypoxia in utero [125, 210]. An increase in the expression of the inflammatory marker, *Tnfα* is associated with a reduction in the methylation of its promoter in offspring from dams with diabetes [144]. Altered *Pgc1α* expression in the skeletal muscle of offspring exposed to protein restriction in utero is associated with the hypermethylation of its promoter [194]. Moreover, in offspring exposed to a maternal high-fat diet, hypermethylation of the *Pparγ* promoter or *Pparγ* gene is observed in the liver and adipose, respectively [113, 199]. In contrast, a reduction in *Pparα* promoter methylation is seen in the liver of offspring exposed to a maternal low-protein diet during gestation [195]*.* In offspring exposed to a maternal protein restriction, the alterations in PGC1α and PPARγ methylation are associated with reduced glucose tolerance and increased circulating triacylglycerols, respectively [194].

The expression of key genes involved in adipocyte differentiation are also impacted by changes in methylation caused by poor maternal environments. For instance, methylation is reduced in association with increased expression of *Zfp423* in the fetus by a maternal obesogenic diet [102]. As mentioned previously, *Zfp423* is involved in adipogenic lineage commitment and changes to its methylation and expression by a maternal obesogenic diet were coupled to advanced adipogenesis in the offspring at weaning and impaired expansion capacity with the introduction of a high-fat diet postnatally [200]. A prenatal low-protein diet is also associated with altered adipocyte differentiation; however, this was associated with increased methylation and expression of the *Igf2* gene in the offspring adipose tissue, as well as elevated adiposity [193]. There was no interactive effect on adiposity, and *Igf2* gene methylation and expression in the offspring exposed to a maternal low-protein diet, by superimposing a high-fat diet postnatally [193].

Identifying the role of changes in genome methylation in animal models of offspring metabolic programming is critical in understanding how to reverse or prevent such programming from occurring. The provision of a methyl donor supplement to the mothers fed a high-fat diet prevents the changes in offspring adipose tissue DNA methylation and expression of genes, including *Fas, leptin* and *Pparγ* [201]*.* The supplement also mitigates the negative metabolic outcomes such as insulin resistance that are induced in the offspring by maternal high-fat diet [201]. Moreover, methyl donors also normalise the methylation level of the leptin gene promoter and reduce offspring leptin concentrations as a result of maternal protein restriction [211]. Changes in the expression and methylation patterns of genes involved in fatty acid metabolism upon methyl-donor group supplementation further demonstrate the importance of methylation as an epigenetic regulator of offspring programming [201, 211]. Maternal intake of folate, a vitamin which is a methyl group donor, has also been studied for its ability to restore normal levels of DNA methylation in offspring tissues that arise as a result of developmental programming. For instance, in a model of maternal protein restriction, folate supplementation largely ameliorates the alterations in genome-wide methylation [212], methylation of the *H19/Igf2* ICR [196] and *Pparα* and *Gr* gene methylation and expression in the offspring [92]. These findings suggest that maternal folate supplementation during pregnancy may have benefit in mitigating negative epigenetic modifications mediating programming effects in offspring tissues as a result of in utero insults.

## Alterations in histone modifications in response to in utero stressors

In the nucleus of eukaryotic cells, DNA is packaged into nucleosomes by wrapping around an octameric histone complex comprised of two copies of H2A, H2B, H3 and H4 histones [222]. Acetylation, methylation, phosphorylation, ubiquitination, sumoylation, glycosylation and ADP-ribosylation occur at the amino termini of histones. Such post-translational modifications alter chromatin structure and the accessibility of transcription factors and DNA binding elements, thus regulating gene expression [223, 224]. The two most common post-translational modifications are acetylation and methylation, whereby acetylation of lysine within the H3 histone activates transcription through inhibition of the secondary and tertiary nucleosome structure and thus, results in chromatin decondensation [225]. Conversely, the methylation of arginine and lysine residues cause chromatin condensation and transcriptional repression [226]. Moreover, post-translational modifications include the addition of marks which activate transcription (H3K9ac, H3K4me3) or marks which repress transcription (H3K27me3, H3K9me2) [227].

Histone alterations appear to mediate the effects of suboptimal gestational environments on offspring pancreatic development and function (Table 3). In offspring exposed to reduced uteroplacental blood flow during gestation, the reduced islet *Pdx-1* expression is associated with a decrease in acetylation of H3 and H4 histones in the fetus and altered methylation of the *Pdx-1*proximal promoter in adult diabetic offspring [117]. These epigenetic modifications in the exposed offspring could be reversed by using a glucagon-like peptide-1 analogue, exendin-4 (Ex-4), which restored histone acetylation and normalised DNA methylation in the islets at the corresponding ages [221]. A maternal low-protein diet also impacts β cell function in adult offspring through changes in histone modifications. In particular, the decreased expression of the transcription factor, *hepatocyte nuclear factor 4-α* (*Hnf4α*) in offspring pancreatic islets, is associated with decreased active and enriched repressive histone marks at regulatory regions of the gene [213].

Histone alterations also play a critical role in the programming of offspring insulin-regulated glucose uptake and metabolism in skeletal muscle. For instance, the alterations in *Glut4* expression in the muscle seen specifically in female rats exposed to a maternal low-protein diet or undernutrition during gestation is related to changes in histone acetylation and methylation at the *Glut4* promoter [72, 94]. Moreover, in the muscle of female and not male offspring, exposure to a maternal low-protein diet during gestation is associated with increased H3 and H4 histone acetylation, as well as expression of the *C/Ebpβ*, a transcription factor involved in the control of skeletal muscle carbohydrate and amino acid metabolism [215]. In female offspring exposed to maternal protein restriction, the alterations in histone modifications at the *Glut4* and *C/Ebpβ* promoters is coupled to increased glycogen content of the skeletal muscle [94]. Together, these studies indicate that changes in histone modifications underlie sex-related differences in the programming of offspring skeletal muscle in response to maternal malnutrition.

Alterations in the expression of genes involved in hepatic development and gluconeogenesis in offspring programmed by a poor intrauterine environment are also mediated by histone modifications. For instance, in offspring of dams with maternal uterine artery ligation, reduced hepatic *Igf1* expression is related to alterations in the H3 histone methylation in both sexes and disrupted H3 acetylation in females specifically [121]. Moreover, the decreased hepatic *Wnt1* expression in pups from dams fed a high-fat diet is linked to altered acetylation of the H4 histone at the *Wnt1* promoter [101]. The liver of pups born from high-fat diet-fed dams also have alterations in histone modifications and these are associated with increased expression of the gluconeogenic genes, *Pck1* [216] and *Pepck* [217]; however, the latter effect is only observed in female offspring [217]. In female offspring exposed to alcohol prenatally, enhanced expression of the *Pten* and *Trb3* by the liver is associated with a reduced acetylation [150]. Furthermore, in offspring from dams fed a low-protein diet, reduced hepatic *Cyp7a1* expression is associated with repressive histone modifications of its promoter and an increase in circulating cholesterol in these offspring [86].

Alterations in histone modifications influence fatty acid metabolism gene expression in the adipose tissue of offspring born to dams with nutritional alterations during gestation. As previously mentioned, an in utero high-fat diet programs alterations in *adiponectin*, *leptin* and *Pparγ* expression in the adipose tissue of offspring. Decreased *adiponectin* and increased *leptin* expression in the offspring adipose tissue is associated with altered levels of acetyl H3K9 and dimethyl H3K9 at their gene promoters [220]. A reduction in *Pparγ2* gene expression in the adipose of offspring born to obese dams is also associated with histone modifications; with adult offspring displaying a reduction in the active mark, H3AC, at the *Pparγ2* promoter [113]. Histone modifications also occur in the adipose tissue of offspring born to dams fed a low-protein diet and given a high-fat diet postnatally. In particular, the reduction in expression of the beige adipocyte marker *fibroblast growth factor 21* (*Fgf21*) in these offspring is associated with an increase in histone methyltransferase G9a [214, 219, 220]. Thus, histone modifications are implicated in the programming of offspring adipose tissue function, lipid metabolism and phenotype.

The reversal or prevention of changes in histone modifications in offspring tissues as a consequence of developmental programming has been achieved through maternally administered interventions. For instance, preconception weight loss in obese mice prevents the changes in lysine acetyltransferase expression and associated reduction in *glucokinase* expression in the fetal liver [218]. In addition, administration of constitutive androstane receptor (CAR) ligand (normally used to improve insulin sensitivity in the context of obesity) to dams fed a high-fat diet during gestation ameliorates the changes in acetyl H3K9 and dimethyl H3K9 that are associated with the *adiponectin* and *leptin* gene promoters and normalises circulating concentrations in the offspring [219]. These data suggest that in a clinical setting, maternal weight loss and ligands like CAR may offer additional benefit, by mitigating the epigenetic modifications and consequent negative metabolic outcomes in the offspring.

## Alterations in small noncoding RNA in response to in utero stressors

Non-coding RNAs are major regulators of gene expression [240–242]. In general, non-coding RNAs have been categorised based on their size and include miRNAs (~ 22 nt), piRNAs (24–30 nt), snoRNAs (60–300 nt) and long non-coding RNA (lncRNAs) (> 200 nt) [243]. miRNAs play a critical role in the epigenetic control of gene expression through regulating DNA methylation and histone modification [244]. Moreover, lncRNAs are involved in genomic imprinting and DNA methylation. For instance, methylation of the paternally inherited allele of the *H19* lncRNA supresses its transcription [245]. Furthermore, lncRNAs affect the function of miRNAs and vice versa. For example, *H19* contains binding sites for the let-7 family of miRNAs and influences their availability [246]. In addition to regulating the expression of genes governing biological processes, non-coding RNAs have been implicated in the development of metabolic disease [247, 248]. However, the role of non-coding RNAs in developmental programming of metabolic disease has been comparatively less studied, with most studies to date looking at the role of miRNAs.

miRNAs are critical in the programming of insulin production in offspring exposed to in utero stress (Table 4). Alterations in the levels of the pancreatic islet-specific miRNA-375, which regulates genes involved in pancreatic islet expansion, occur in response to insulin resistance and contribute to the development of type 2 diabetes in vivo [248–251]. The abundance of miRNA-375 is higher in the islets of rat foetuses and adult offspring exposed to a maternal low-protein diet in utero [229]*.* Elevated miRNA-375 levels are associated with impaired islet *Pdx-1* expression, proliferation and insulin secretion and the development of diabetes in these offspring [229]. Moreover, normalizing miR-375 levels in the islets derived from adult offspring of protein-restricted dams restores their capacity to secrete insulin and proliferate in vitro [229]. Changes in miRNA expression are also implicated in the development of peripheral insulin resistance in offspring exposed to an obesogenic diet in utero. In particular, the decrease in the insulin signalling protein, IRS1, in the adipose tissue of offspring exposed to a maternal high-fat diet is linked to an upregulation of *miR-126* [233].

Pathological processes relating to metabolic dysfunction such as altered lipid storage and subsequent lipotoxicity and insulin resistance are also regulated by miRNAs in the adipose tissue. For instance, an upregulation of *miR-483-3p* is observed in the white adipose of adult rats exposed to a maternal low-protein diet [230]. In vitro, *miR-483-3p* inhibits adipocyte differentiation and lipid accumulation through reducing *growth differentiation factor-3* (*Gdf3*) expression, and in turn, *Fabp4* and *Pparγ* [230]. Defects in the formation of brown adipose tissue in the fetus may also contribute to the later development of poor lipid handling and genesis of metabolic syndrome as a result of poor conditions during in utero development. For instance, defects in brown tissue formation, mitochondriogenesis and mitochondrial capacity in fetuses from obese mouse mothers is associated with an upregulation of *miR-204-5p,* which negatively regulates *Pgc1α* and *Sirt1* expression [239]. Thus, miRNAs are implicated in key metabolic alterations to offspring adipose tissue, namely reduced browning and increased lipid accumulation.

Alterations in lipid metabolism in the liver of offspring exposed to poor gestational conditions may also be partly induced by alterations in miRNA levels. In the liver of adult offspring of mothers given a high-fat diet or exposed to caloric restriction during gestation, *miR-122* levels are decreased in association with increased expression of genes involved in lipid synthesis (e.g, *Pparα, Pparβ, Scd1*) and fatty acid oxidation (e.g, *Pgc1α* and *Cpt-1α*) and an associated reduction in hepatic triglycerides [228, 234–236]. In offspring from mothers fed a high-fat diet during gestation, the reduction in hepatic *miR-122* expression is not prevented by cross-fostering to mothers fed a control diet during lactation [237]. In offspring of mothers given a low-protein or high-calorie diet during gestation, the hepatic inflammation observed appears to be mediated by dysregulated expression of several miRNAs that contribute to the increased expression of *Tnfα* and *Il-6* genes [232, 238]. Together, these studies highlight that miRNAs may serve as valuable therapeutic targets to prevent the developmental programming of metabolic syndrome in offspring exposed to adverse gestational environments.

Whilst the role of other of non-coding RNAs species in the programming of offspring metabolic disease has been less studied, lncRNAs have been shown to regulate several metabolic pathways including gluconeogenesis and fatty acid metabolism [252, 253]. Moreover, in offspring exposed to a low-protein diet in utero, defective β cell expansion is associated with the decreased expression of the lncRNA, *H19* [231]. In part, the association between *H19* expression and β cell expansion is thought to be related to loss of *H19*-mediated inhibition of *Let-7*, a negative regulator of PI3K-AKT signalling [231]. Together, these results indicate the importance of lncRNA in influencing metabolic processes in the programming of offspring phenotype.

## Conclusions and future directions

**Fig. 1:**
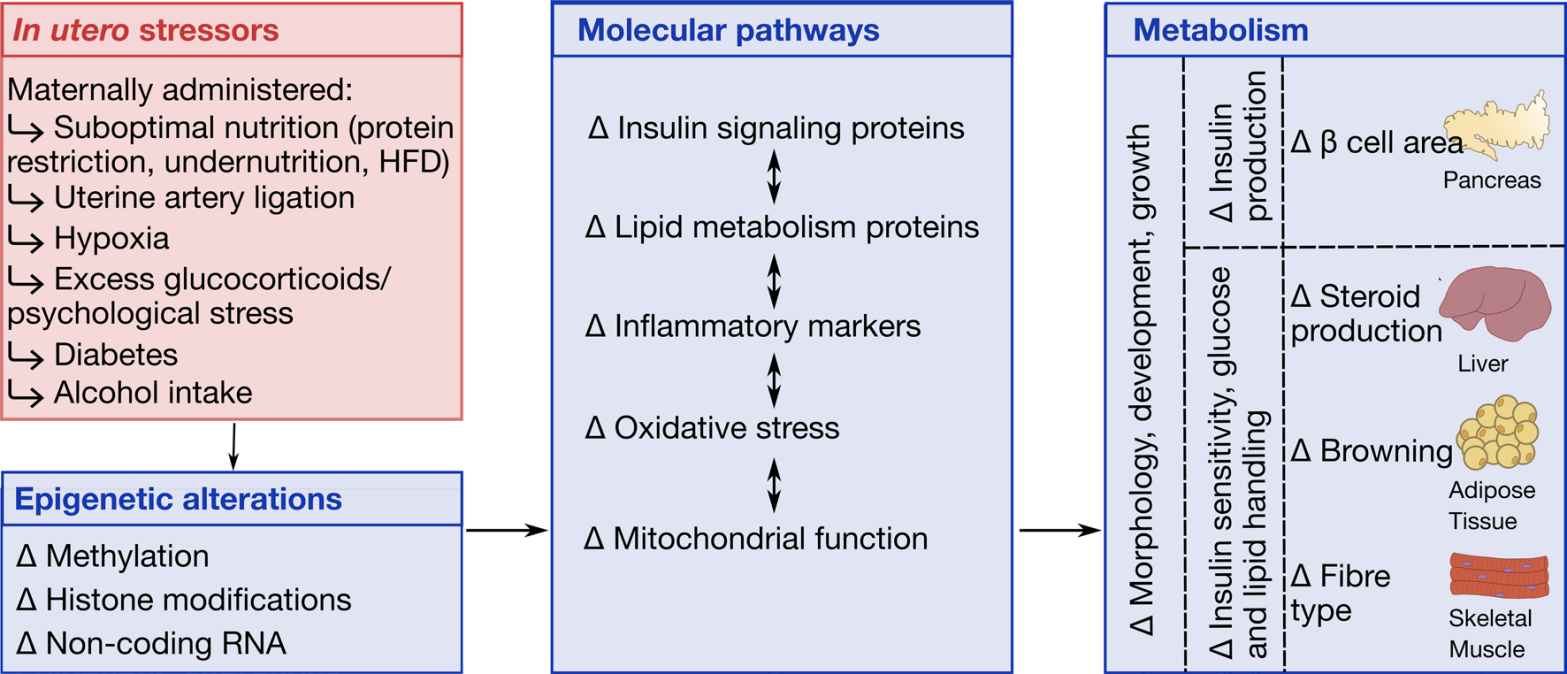
The effect of in utero stressors on epigenetic, gene expression and structural alterations in metabolic organs of offspring. Δ, change in; HFD, high-fat diet

This review focused on the contribution of suboptimal maternal environments (in utero stress) to the developmental programming of offspring metabolic health via changes in key metabolic tissues in the first generation. Data from rodent models show that in utero stress can lead to epigenetic changes in key metabolic organs, which impact growth, morphology, insulin sensitivity, nutrient handing, metabolism and inflammation with consequences for the metabolic physiology of the offspring (Fig. 1). However, the placenta is the interface between the mother and fetus and mediates maternal insults imposed through the regulation of fetal nutrient provision. Thus, although not addressed in the current review, the placenta will have contributed to the programming of offspring metabolic disease as a result of in utero stress (reviewed in [254–256]). Moreover, half of the placental and fetal genome derives from the father. A significant paternal contribution to the developmental programming of offspring metabolism has recently come to light, with paternal exposure to alcohol or a high-fat or low-protein diet also programming adult offspring metabolism [257–259]. The mechanisms by which this occurs have been reviewed elsewhere [260–264] and above all highlight the need to also consider paternal environmental exposures when elucidating the mechanisms underlying the programming of metabolic disturbances in the offspring. Furthermore, the transmission of metabolic dysfunctions has been observed across subsequent generations even in the absence of further insults and normal environmental conditions via epigenetic processes (discussed previously; [265–267]). Moreover, in utero stressors can also program defects in the function in other systems that may interact with the metabolic system of the offspring, including the reproductive, neuroendocrinological, microbiome and vasculature systems [268–274]. To date, the contribution of alterations in the methylome of fetal and adult tissues to the programming of offspring metabolic phenotype has been most widely studied although other epigenetic factors, such as histone modifications and non-coding RNA are also emerging as key players in this process and require further investigation. However, other mediators such as modification to mitochondrial DNA (which are normally inherited from the mother), de novo point mutations in the germline or somatic cells and vertical transmission of microorganisms to offspring can contribute to the developmental programming of metabolic syndrome postnatally [116, 275–277].

Undeniably, studies in rodent models have been useful in showing causation between specific gestational insults and offspring outcomes by selectively manipulating a variable in a controlled way. Indeed, the studies in this review illustrate that in many instances a variety of maternal manipulations imposed on rodents act to perturb the same metabolic pathways, as postulated by the gatekeeping hypothesis [278]. However, it is important to note that in some instances, there are inconsistencies in the changes observed for molecular pathways in the same, let alone different rodent model of in utero stress, making it challenging to draw firm conclusions on the commonality of pathways involved. Moreover, studies have typically been limited by not considering offspring sex or only studying the outcomes of in utero stress on the male offspring (due to challenges faced by the need to control for the influence of the oestrus cycle on metabolic function in females). Indeed, in the few studies that have studied both female and male offspring, sexually-dimorphic metabolic outcomes of developmental programming have frequently been observed and may be attributed to a variety of factors, including the effect of sex chromosome-linked genes in the placenta, as well as sex differences in the temporal development of metabolic organs and fetal and adult levels of hormones like corticosterone/cortisol and sex steroids [279–283]. Thus, to further address the molecular mechanisms underlying the developmental programming of offspring metabolic health, we recommend that both male and female offspring are studied in future work. Differences in strain, husbandry, control diet composition, severity and duration of the insult, cross fostering techniques and standardisation of litter size postnatally, are also parameters that have been shown to influence metabolic health outcomes [284–288]. For instance, methods to impose “maternal stress” in rodent models of programming range from saline injection, to swimming and physical restraint, which are likely to have differing impacts on raising endogenous corticosterone/cortisol in the mother. Whilst in most studies, the maternal insult is confined to the gestational period, in some instances, insults are continued into the lactational period. As such it is difficult to discriminate whether the programming of offspring metabolic physiology is occurring during gestation or lactation and studies should be designed to more directly address this in future work. Furthermore, future studies should be aimed at more accurately depicting clinical settings, where changes in prenatal environment can be due to a variety of causes, operating at different times in gestation, which may together impact and drive resulting offspring metabolic outcomes. Moreover, future research may also benefit from developing diagnostic criteria to grade metabolic health at certain ages and stages of the disease process in rodent species, as is done in a clinical setting. However, taken together, rodent models have undoubtedly provided significant insight into understanding the ontogeny and molecular mechanisms by which offspring metabolic health may be programmed by exposure to in utero stress. Further, with their continued use, rodent models may provide the knowledge needed to improve or prevent the propagation of metabolic diseases that are reaching epidemic proportions in many countries across the world.
